# Assembly and dynamics of the U4/U6 di-snRNP by single-molecule FRET

**DOI:** 10.1093/nar/gkv1011

**Published:** 2015-10-25

**Authors:** John W. Hardin, Chandani Warnasooriya, Yasushi Kondo, Kiyoshi Nagai, David Rueda

**Affiliations:** 1 Department of Medicine, Section of Virology, Imperial College London, London W12 0NN, UK; 2 Single Molecule Imaging Group, MRC Clinical Sciences Centre, Imperial College London, London W12 0NN, UK; 3 MRC Laboratory of Molecular Biology, Francis Crick Avenue, Cambridge CB2 0QH, UK

## Abstract

In large ribonucleoprotein machines, such as ribosomes and spliceosomes, RNA functions as an assembly scaffold as well as a critical catalytic component. Protein binding to the RNA scaffold can induce structural changes, which in turn modulate subsequent binding of other components. The spliceosomal U4/U6 di-snRNP contains extensively base paired U4 and U6 snRNAs, Snu13, Prp31, Prp3 and Prp4, seven Sm and seven LSm proteins. We have studied successive binding of all protein components to the snRNA duplex during di-snRNP assembly by electrophoretic mobility shift assay and accompanying conformational changes in the U4/U6 RNA 3-way junction by single-molecule FRET. Stems I and II of the duplex were found to co-axially stack in free RNA and function as a rigid scaffold during the entire assembly, but the U4 snRNA 5′ stem-loop adopts alternative orientations each stabilized by Prp31 and Prp3/4 binding accounting for altered Prp3/4 binding affinities in presence of Prp31.

## INTRODUCTION

Eukaryotic genes are often organized as a series of coding regions (exons) separated by intervening non-coding regions (introns). Introns are excised from precursor messenger RNA (pre-mRNA), while exons are spliced together to form a mature mRNA with a continuous protein coding sequence by a massive RNA-protein machine called the spliceosome (1,2). The major components of the spliceosome are five small nuclear ribonucleoprotein particles, (U1, U2, U4, U5 and U6 snRNPs) each containing one of the five spliceosomal U-type snRNAs (U1, U2, U4, U5 and U6 snRNAs), seven Sm or LSm proteins and other particle-specific proteins. These snRNPs assemble in an ordered manner onto pre-mRNA substrates together with non-snRNP proteins. Firstly, the U1 and U2 snRNPs associate with the 5′ splice site and the highly conserved branch point sequence located within the intron to be excised, respectively (3). This U1/U2/pre-mRNA complex is referred to as the pre-spliceosome or complex A. Next, a tri-snRNP particle composed of the U4/U6 and U5 snRNPs associates with the pre-spliceosome, forming the pre-catalytic spliceosome or complex B. This association results in a significant structural rearrangement of the U4/U6.U5 tri-snRNP particle leading to the catalytically active spliceosomal complex B*, upon release of the U1 and U4 snRNPs and formation of a U2/U6 snRNA pair. The first catalytic step of splicing then involves pre-mRNA cleavage at the 5′ splice site and ligation of the 5′ end of the intron to the branch site resulting in a lariat intron structure similar to the intermediate of the group II self-splicing intron (4–6). Structural rearrangements at this stage yield complex C, which then catalyzes cleavage at the 3′ splice site and the formation of mature mRNA through ligation of the 5′ and 3′ exons.

**Figure 1.**
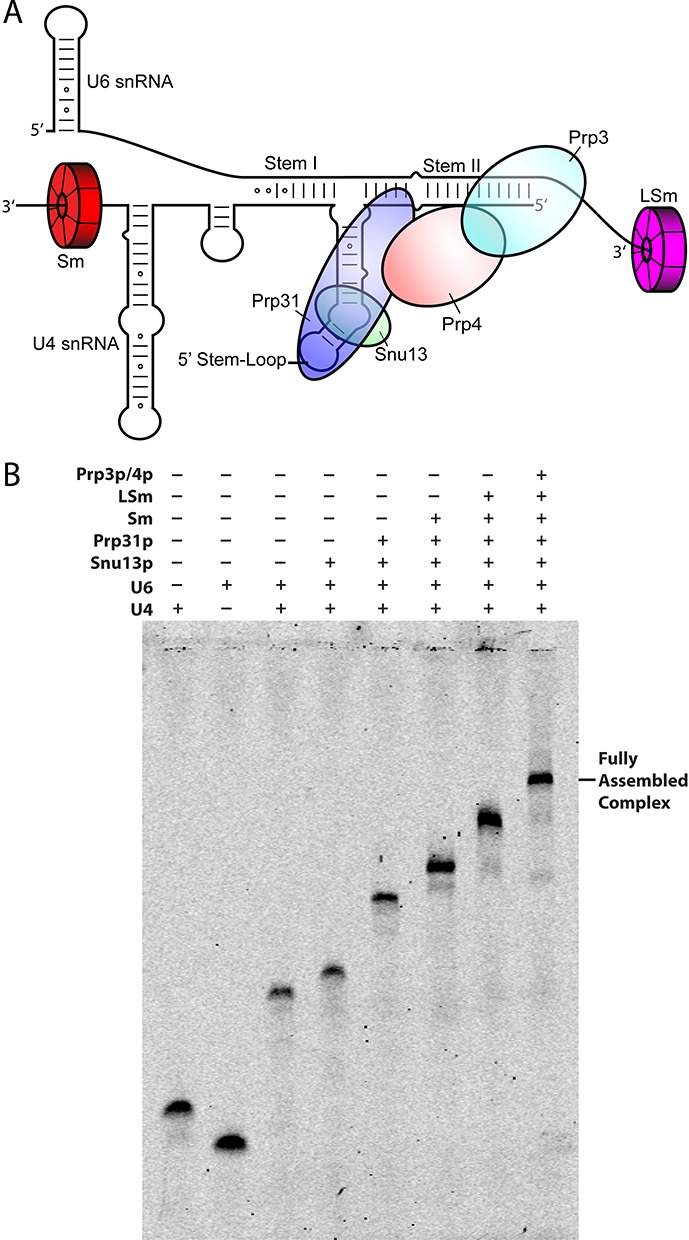
(**A**) Secondary structure representation of the yeast U4/U6 di-snRNP. Each snRNP protein is color-coded and labeled accordingly. (**B**) Stepwise assembly of the full U4/U6 di-snRNP under sub-stoichiometric conditions followed by electrophoretic mobility shift assay (EMSA). Consecutive binding of each protein results in complete gel shifts, indicating step-wise assembly of the snRNP.

The *Saccharomyces cerevisiae* U4/U6 di-snRNP is composed of U4 and U6 snRNAs, and 18 proteins (Figure 1A): Snu13, Prp31, Prp3, Prp4, seven Sm and seven LSm proteins (7–9). The pre-formed LSm protein ring binds to the binding sequences at the 3′ ends of the U6 snRNAs (10–13), and three Sm protein sub-complexes, namely SmB-SmD3, SmD1-SmD2 and SmE-SmF-SmG, assemble around the Sm sequence near the 3′ end of U4 snRNA (14–16). Snu13 binds to the kink turn (k-turn) motif in the 5′ stem-loop of U4 snRNA (Figure 1A) and facilitates Prp31 binding (8,17,18). The structure of a ternary complex comprising human Snu13, Prp31 and 5′ stem-loop of U4 snRNA has been reported (19). Prp3 and Prp4 are known to be the only U4/U6 di-snRNP specific proteins (20–22). They form a dimer prior to binding around stem II and 5′ stem-loop of U4/U6 duplex (8,23,24). However, little is known regarding the global structure of the U4/U6 di-snRNP. Currently, the only global structural information is from a low resolution (∼40 Å) EM structure revealing a large and a small domain connected by a thin bridge (25). Although this study provides some basic low-resolution information about the structure of the U4/U6 di-snRNP and how it associates within the tri-snRNP, the relative orientation of the helices of the 3-way junction (between stem I, stem II and the 5′ stem-loop) and the global structure of the U4/U6 snRNA duplex in the presence of its associated proteins remains structurally unresolved. A modeling study done by Lescoute and Westhof (26), has categorized RNA 3-way junctions with two coaxially stacked helices into three groups based on the length of the linkers connecting the helices. It has been proposed that U4/U6 snRNA duplex belongs to the B family, with stem I and 5′ stem-loop of U4 snRNA are coaxially stacked.

We have over-expressed all the protein components of the yeast U4/U6 snRNP using *E. coli* and yeast expression systems. This has allowed us to determine the affinity of proteins upon stepwise addition of protein during the complete assembly of the U4/U6 di-snRNP. Proteins capable of directly interacting with the RNA were systematically examined for their ability to nucleate further assembly with apparent binding affinities reported for all components at each stage. In this study, we have developed a protocol to reconstitute a complete di-snRNP *in vitro*. We then investigated the global conformation and conformational changes in the U4/U6 snRNA duplex three-way junction with step-wise protein assembly by employing single-molecule fluorescence resonance energy transfer (smFRET), a powerful technique to characterize, in real time, conformational and folding dynamics of RNA complexes otherwise hidden in ensemble averaged studies (27–29). Taken together, our data provide new insight into the global conformation and assembly of the U4/U6 di-snRNP.

## MATERIALS AND METHODS

### Protein cloning, expression and purification

#### Snu13 expression and purification

The Snu13 gene was PCR amplified from *Saccharomyces cerevisiae* genomic DNA and cloned by standard techniques into a modified pRK172 vector placing a TEV protease cleavable hexa-histidine tag at the N-terminus. BL21(DE3)-RIL CodonPlus cells (Stratagene) were transformed and cultured in 2xTY media with 35 μg/ml chloramphenicol and 50 μg/ml ampicillin at 37°C. Expression was induced with 0.5 mM IPTG at an OD at 600 nm of 0.6. The cells were spun down after 6 h, resuspended in 20 mM Tris-HCl (pH 7.4), 500 mM NaCl, 500 mM urea, 25 mM imidazole, 10 mM β-mercaptoethanol, with complete protease inhibitor mixture (Roche) and lysed by sonication. The cell lysate was cleared by centrifugation and the supernatant was applied to a Ni-NTA agarose (Qiagen) column, washed with 20 mM Tris-HCl (pH 7.4), 1 M NaCl, 500 mM urea, 25 mM imidazole, 10 mM β-mercaptoethanol, and eluted under these buffering conditions with a linear gradient of imidazole to 1 M over 400 ml. Snu13 containing fractions were treated with TEV protease during dialysis against 20 mM Tris-HCl (pH 7.4), 500 mM NaCl, 500 mM urea, 25 mM imidazole, 10 mM β-mercaptoethanol, at room temperature and then reapplied to Ni-NTA resin to remove the tag. Snu13 was dialyzed into 20 mM Tris-HCl (pH 7.4), 50 mM NaCl, 10 mM β-mercaptoethanol, and the protein was loaded onto an SP sepharose column (GE Healthcare) and eluted with a linear gradient of NaCl to 1 M over 400 ml. Pooled fractions were dialyzed against 10 mM potassium phosphate (pH 7.2), 50 mM NaCl, applied to a hydroxyapatite column (Biorad), and eluted by a gradient of ammonium sulfate to 6% (w/w). Snu13 containing fractions were concentrated and buffer exchanged into 1 mM Tris-HCl (pH 7.4).

#### Prp31 expression and purification

The Prp31 coding sequence was PCR amplified from *Saccharomyces cerevisiae* genomic DNA and cloned into a pGEX 6P-1 vector for expression with an N-terminal GST tag cleavable by Prescission protease and a non-cleavable C-terminal octa-histidine tag. BL21(DE3)-RIL CodonPlus cells (Stratagene) were transformed and cultured in 2xTY media with 35 μg/ml chloramphenicol and 50 μg/ml ampicillin at 20°C. Expression was induced with 0.5 mM IPTG at an OD at 600 nm of 0.6. The cells were spun down after 6 h, resuspended in 20 mM Tris-HCl (pH 7.4), 500 mM NaCl, 500 mM urea, 10 mM β-mercaptoethanol, with complete protease inhibitor mixture (Roche), lysed by sonication, and cleared by centrifugation. The cleared lysate was loaded onto a GST column (GE Healthcare), washed with 20 mM Tris-HCl (pH 7.4), 1 M NaCl, 500 mM urea, 10 mM β-mercaptoethanol, and eluted with a gradient of glutathione to 25 mM. Prp31 containing fractions were applied to a Ni-NTA column in 20 mM Tris-HCl (pH 7.4), 500 mM NaCl, 500 mM urea, 25 mM imidazole, 10 mM β-mercaptoethanol, column washed with 20 mM Tris-HCl (pH 7.4), 1 M NaCl, 500 mM urea, 25 mM imidazole, 10 mM β-mercaptoethanol, and protein eluted with 20 mM Tris-HCl (pH 7.4), 500 mM NaCl, 500 mM urea, 1 M imidazole, 10 mM β-mercaptoethanol. The eluate was treated with 3C Prescission protease (GE Healthcare) at room temperature while dialyzing against 20 mM Tris-HCl (pH 7.4), 500 mM NaCl, 500 mM urea, 10 mM β-mercaptoethanol. After 5 h incubation at room temperature, the solution was applied to a GST column to remove the GST tag. The flow-through was dialyzed against 20 mM Tris-HCl (pH 7.4), 150 mM NaCl, 10 mM β-mercaptoethanol, applied to a SP-sepharose column (GE Healthcare), and protein eluted with a linear gradient of NaCl to 1 M over 300 ml. The protein was then dialyzed against 10 mM potassium phosphate, 250 mM KCl, and purified on a hydroxyapatite column (Biorad) with a 150 ml linear gradient to 12% ammonium sulfate. The eluate was concentrated and buffer exchanged to 10 mM NaHEPES (pH 6.8), 300 mM NaCl.

#### Prp3/4 expression and purification

The Prp3 and Prp4 coding sequences were PCR amplified from *Saccharomyces cerevisiae* genomic DNA. Prp3 was cloned into a modified pUC 18 vector that placed a TEV protease cleavable octa-histidine tag at the C-terminus and contained a pGGAP promoter and 3′-UTR (30). The Prp4 coding sequence was likewise cloned into a similar vector lacking the affinity tag. The cassettes containing the pGGAP promoter, Prp3 or Prp4, and the 3′-UTR, were excised and cloned into a single pRS426 vector (Invitrogen). The pRS vector containing Prp3 and Prp4 coding sequences was transformed into competent BCY123 yeast cells (MATα, Can1, ade2, trp1, Ura3–52, his3, leu2–3, 112, pep4::his+, prb1::leu2+, bar1::HisG+, lys2::pGAL1/10-GAL4+) by the lithium acetate method (31), plated on -Ura plates, and incubated at 30°C for 2 days. A single colony was inoculated into 50 ml of -Ura YM media with 2% raffinose and grown at 30°C for 24 h as a pre-culture. 12 l of -Ura YM media with 2% raffinose were innoculated and grown at 30°C to an OD 600 of 0.8 before induction with 2% galactose. The cells were spun down after overnight growth at 30°C and lysed using a 6870 freezer/mill (SPEX), clarified by centrifugation, and applied to a Ni-NTA column as for other proteins used in this study. The combined fractions were applied to a hydroxyapatite column pre-equilibrated with 20 mM sodium phosphate (pH 7.2), 200 mM NaCl, 10 mM β-mercaptoethanol, and eluted by a gradient of sodium phosphate to 200 mM. The eluate was dialyzed against 20 mM Tris-HCl (pH 7.4), 300 mM NaCl, 10 mM β-mercaptoethanol, and applied to a Resource-Q column (GE Healthcare) after dilution with chilled water to reduce the concentration of NaCl to 150 mM immediately before applying to the column. Protein was eluted under these buffering conditions with a linear gradient of NaCl to 1 M. The pooled fractions were concentrated and buffer-exchanged to 20 mM Tris-HCl (pH 7.4), 500 mM NaCl, 10 mM β-mercaptoethanol, applied to a Superdex-200 gel filtration column, and concentrated for storage at −80°C.

#### Sm protein expression and purification

All Sm protein genes (SmB, D1, D2, D3, E, F and G) were PCR amplified from *Saccharomyces cerevisiae* genomic DNA. Sm proteins were expressed and purified as previously described (16,32).

#### LSm2–LSm8 expression and purification

All LSm protein genes (LSm2–LSm8) were PCR amplified from *Saccharomyces cerevisiae* genomic DNA and cloned into a modified pUC18 vector containing the pGGAP promoter and 3′-UTR. The LSm8 gene was cloned so as to place a TEV protease cleavable CBP tag at the C-terminus and the LSm5 gene was cloned so as to place a TEV protease cleavable octa-histidine tag at the C-terminus. The rest of LSm protein genes (LSm2, LSm3, LSm4 (1–106), LSm6 and LSm7) were untagged. The expression cassettes including pGGAP promoter, LSm protein gene and 3′-UTR, were cut out and cloned into pENTR3C vectors (Invitrogen) creating the pENTR3874 and pENTR265 vectors containing LSm3, LSm8-CBP, LSm7, LSm4, and LSm2, LSm6, LSm5-His, respectively. The final expression vectors were then made by the Gateway cloning reaction using the clonase II enzyme (Invitrogen) to promote LR recombination between the pENTR3C vector containing multiple LSm protein genes and either pRS424 or pRS426 vectors that had been modified to contain DNA sequences for ccdB and chloramphenicol resistance flanked by the required attR1 and attR2 sites derived from the pDEST8 vector (Invitrogen). This resulted in pRS426LSm3874 and pRS424LSm265 vectors. These vectors were co-transformed into BCY123 using the lithium acetate method (33). Transformants were selected on –Ura –Trp plates and a single colony was inoculated into 50 ml of -Ura -Trp YM media with 2% raffinose and grown at 30°C for 24 h as a pre-culture. 12 L of -Ura -Trp YM media with 2% raffinose were inoculated and grown at 30°C to an OD 600 of 0.8 before induction with 2% galactose. The cells were spun down and resuspended in 10 mM Tris-HCl (pH 8.0), 1 mM magnesium acetate, 1 mM imidazole, 2 mM CaCl_2_, 500 mM NaCl and 10 mM β-mercaptoethanol, with complete protease inhibitor cocktail (Roche), lysed using a 6870 freezer/mill (SPEX) and clarified by centrifugation. The supernatant was incubated with calmodulin resin at 4°C overnight. Protein was eluted with 10 mM Tris-Cl (pH 8.0), 1 mM Magnesium acetate, 1 mM imidazole, 500 mM NaCl, 10 mM β-mercaptoethanol and 2mM EGTA. The fractions containing the LSm 2–8 complex were dialyzed against 20 mM Tris-Cl (pH 7.4), 500 mM NaCl, 500 mM urea, 25 mM imidazole, 10 mM β-mercaptoethanol, applied to a Ni-NTA column, and eluted by an imidazole gradient to 500 mM. Eluted protein was dialyzed against 10 mM potassium phosphate (pH 7.2), 300 mM KCl, 10 mM β-mercaptoethanol, applied to a hydroxyapatite column (Bio-Rad), and eluted with an ammonium sulphate gradient to 12%. The protein containing fractions were dialyzed against 20 mM Tris-HCl (pH8.0), 300 mM NaCl, 10 mM β-mercaptoethanol, and complex further purified through a Mono-Q column (GE Healthcare) in 20 mM Tris-HCl (pH 8.0), 100 mM NaCl, 10 mM β-mercaptoethanol with a linear gradient of 20 mM Tris-HCl (pH 8.0), 1 M NaCl, 10 mM β-mercaptoethanol producing a homogeneous main peak followed by several minor peaks found to be dimeric and trimeric species of the LSm2–LSm8 complex by native mass spectrometry. This final material was found to be very stable in solution capable of withstanding concentrations in excess of 20 mg/ml under low salt conditions (<100 mM KCl).

### RNA cloning, transcription, purification and labeling

RNAs were transcribed as Hammerhead (HH) ribozyme-RNA-Hepatitis delta virus (HDV) ribozyme fusions and purified on acrylamide gels after co-transcriptional HH and HDV self-cleavage at the insert–ribozyme junction.

#### Cloning of HH-RNA-HDV fusions

The target sequence for *in vitro* transcription was PCR amplified using synthetic DNA primers to generate the T7 promoter-HH-RNA construct and then cloned by standard techniques into a modified pUC19 vector containing the HDV sequence by restriction/ligation. The same strategy, using different oligonucleotide sequences, was used to generate both the U4 and U6 snRNAs. The accuracy of the inserts and ribozyme placement was confirmed by DNA sequencing.

#### In vitro RNA transcription and purification

Plasmids for *in vitro* RNA transcription were prepared and purified by CsCl ultracentrifugation. The plasmids were linearized 3′ of the HDV ribozyme and RNA was transcribed by standard methods (34). The RNA product was purified on 8% polyacrylamide denaturing gels run with 8 M urea in TBE [89 mM Tris–borate, 2 mM EDTA (ethylenediaminetetraacetic acid), pH 8.3], visualized by UV shadowing, excised from the gel, electroeluted from the acrylamide, and exchanged and concentrated in water.

#### Fluorescein labeling of RNA

RNA used in assembly studies was labeled with fluorescein at the 3′- end. A total of 100 μg of RNA was used in a 130 μL reaction consisting of 40 mM Na-MES (pH 6.0), 10 mM MgCl_2_, 5 mM DTT, 10 U CIP and 200 U T4 PNK (35). This mixture was incubated at 37°C for 3 h after which time the RNA was phenol/chloroform extracted and ethanol precipitated. The 3′ vicinal diol was oxidized by resuspending the RNA in 100 μL of freshly made oxidation solution [0.1 M sodium periodate and 0.1 M sodium acetate (pH 5.0)] and incubated at room temperature for 1.5 h in the dark (36). The reaction was quenched by the addition of 11 μL of 2.5 M KCl, placed on ice for 10 min, and the resultant insoluble KIO4 pellet was removed by a brief centrifugation. A thiosemicarbazide derivative of fluorescein (100 mM in DMSO) was added to a final concentration of 50 mM and incubated at room temperature for 4 h (37). Three phenol/chloroform extractions were performed to remove most of the free fluorophore, the labeled RNA ethanol precipitated, and gel purified by denaturing PAGE.

#### U4/U6 snRNA duplex formation

For substoichiometric electrophoretic mobility shift assays, U4 snRNA labeled with fluorescein at the 3′ end was mixed with unlabeled U6 snRNA to a final concentration of 1 μM and 2 μM, respectively, in 10 mM K-HEPES (pH 7.5), 100 mM KCl. The mixture was heated to 90°C and slow-cooled to 4°C at −0.03°C/s. The RNA duplex was then gel purified on a native gel at 4°C, band excised, RNA eluted by the ‘crush and soak’ method into 10 mM K-HEPES (pH 7.5), 100 mM KCl, and concentrated in an ultra-centrifugation filter (Amicon).

### Electrophoretic mobility shift assays

#### Substoichiometric assembly analysis

Direct binding electrophoretic mobility shift assay (EMSA) experiments were performed with samples containing 2 nM fluorescein labeled RNA and protein typically within the range ∼0.3 pM to 2.5 μM in an EMSA sample buffer consisting of 10 mM K-HEPES (pH 7.5), 100 mM KCl, 0.01% NP-40, 20 μg *E. coli* tRNA in a volume of 100 μl. For step-wise assembly (Table 1), protein concentrations for pre-assembled components were Snu13 (200 nM), Prp31 (120 nM), Sm proteins (64 nM) and LSm proteins (240 nM). Reactions were allowed to equilibrate on ice for 60 min before loading on native polyacrylamide gels (4% at 37.5:1 acrylamide:bis-acrylamide) run in 0.5X TBE buffer at 4°C. Gels were imaged on a Typhoon variable-mode scanner and the signals in the gel bands corresponding to protein bound and unbound RNA were integrated. Parameters in the following function were fit to the data for fraction of RNA bound versus protein concentration:

\begin{equation*} \theta = \left[ {\frac{{a - b}}{{1 + \left( {\frac{{Kd,app}}{{[protein]}}} \right)^n }}} \right] + b \end{equation*}

where θ is fraction bound, K_d, app_ is the apparent dissociation constant, a is the upper baseline, b is the lower baseline and n is the Hill coefficient. At least two gel shifts were performed for each sample and associated error is reported as one standard deviation from the mean (Table 1). The shifts in Table 1 are for entirely wild-type components with the exception of the LSm complex wherein LSm4 was truncated (amino acids 1–106) so as to remove the C-terminal region absent from the human homolog and predicted to be disordered (DISOPRED) (38) and a truncated Sm complex was used (SmB 1–105).

**Table 1.** Apparent binding affinities (K_d, app_) and Hill coefficients for step-wise *in vitro* assembly of the *Saccharomyces cerevisiae* U4/U6 di-snRNP as determined by EMSA. Confidence intervals are one standard deviation from the weighted mean.

Components	Titrated Component	K_d, app_	Hill Coefficient
U4/U6	Snu13	17 ± 1 nM	1
U4/U6	Prp31	243 ± 16 nM	1.9 ± 0.2
U4/U6	Sm	89 ± 4 nM	3.5 ± 0.4
U4/U6	LSm	5 ± 0.2 nM	1.7 ± 0.1
U4/U6	Prp3/4	57 ± 2 nM	2.6 ± 0.3
U4/U6/Snu13	Prp31	50 ± 4 nM	2.9 ± 0.5
U4/U6/Snu13	Sm	92 ± 9 nM	2.2 ± 0.4
U4/U6/Snu13	LSm	26 ± 1 nM	3.3 ± 0.3
U4/U6/Snu13	Prp3/4	88 ± 8 nM	3.4 ± 1.0
U4/U6/Snu13/Prp31	Sm	108 ± 9 nM	2.3 ± 0.4
U4/U6/Snu13/Prp31	LSm	98 ± 34 nM	1
U4/U6/Snu13/Prp31	Prp3/4	417 ± 43 nM	1.5 ± 0.2
U4/U6/Snu13/Prp31/Sm	LSm	154 ± 15 nM	1
U4/U6/Snu13/Prp31/Sm	Prp3/4	557 ± 75 nM	1
U4/U6/Snu13/Prp31/Sm/LSm	Prp3/4	20 ± 1 nM	1.6 ± 0.1

Concentrations used in Supplementary Figure S6A were U4 (4 nM), U6 (4 nM), U4/U6 snRNA duplex (4 nM), Snu13 (200 nM), Prp31 (120 nM), and the shift with LSm was conducted with 2-fold dilutions and a maximum concentration of 1 μM. Supplementary Figure S6B had H46 hybrid RNA (10 nM), Snu13 (100 nM), Prp31 (150 nM) and the shift with LSm was conducted with 2-fold dilutions and a maximum concentration of 500 nM. Figure 1B had U4 (4 nM), U6 (4 nM), U4/U6 snRNA duplex (4 nM), Snu13 (180 nM), Prp31 (120 nM), Sm proteins (64 nM), LSm proteins (240 nM) and Prp3/4 (500 nM).

### Single-molecule FRET

#### Sample purification and labeling

Three U6 RNA strands and one U4 RNA strand were utilized for the single-molecule experiments to study the orientation of three helices (Supplementary Figure S1C and Table S1). The U4 and U6-II strands were purchased from Dharmacon, whereas U6-I and U6-III strands were purchased by Keck Foundation Resource Laboratory at the Yale University School of Medicine.

The 2′-hydroxyl protective groups on all four RNA strands were removed and the RNAs were purified as previously described (39,40). The RNAs were purified by denaturing gel electrophoresis (20% wt/vol polyacrylamide and 8 M urea) and diffusion elution against elution buffer (0.5 M NH_4_OAc and 0.1 mM EDTA) overnight at 4°C, followed by chloroform extraction, ethanol precipitation and C8 reverse-phase HPLC. The C6 amino modifier in U6-II was labeled with Cy3 (GE Healthcare), while the C6 amino modifier in U6-III and a 5-LC-NU internal amino modifier in U4 were labeled with Cy5 (GE Healthcare) in labeling buffer (100 mM Na_2_CO_3_, pH 8.5) overnight at 27°C. The labeled RNAs were further purified by ethanol precipitation and reverse-phase HPLC. RNA concentrations were measured by UV-Vis absorbance at 260 nm.

#### Single-molecule experiments

Single-molecule experiments were performed as described (39,41). Two RNA strands (2 μM U4 and 2 μM U6-I, U6-II or U6-III) in standard buffer [10 mM Tris-HCl (pH 7.4), 100 mM NaCl] were heated at 94°C for 45 s and annealed by cooling to room temperature over 20 min. The annealed, biotinylated, fluorophore-labeled complex was then diluted to 10 pM and immobilized on a quartz slide via a biotin-streptavidin interaction to generate a surface density of ∼0.1 molecules/μm^2^. An oxygen-scavenging system (OSS) consisting of 5 mM protocatechuic acid (PCA) and 0.1 μM protocatechuate-3, 4-dioxygenase (PCD) was used to reduce photo-bleaching (42). In protein binding experiments, the measurements were obtained in the presence Snu13, Prp31 or Prp3/4.

The donor fluorophores were excited in a home-built total internal reflection microscope with a laser (532 nm, 2 mW, Laser 2000). The donor and acceptor emission were separated using appropriate dichroic mirrors (635DCXR, Chroma) and detected as two side-by-side images on a back-illuminated electron-multiplied CCD camera (Andor I-Xon Ultra 897) (39,41,43). The individual donor (I_D_) and acceptor (I_A_) intensities of optically resolved single molecules (characterized by single-step photo-bleaching) were measured by integration of their relative spot intensities and used to calculate the apparent FRET efficiency as FRET = I_A_/(I_A_ + I_D_), and followed in real time for each molecule. Resulting time trajectories were then separated based on the FRET value and time binned to draw FRET histograms, which represent the frequency of the population at a particular FRET value. Average FRET values for each population were determined by fitting the histograms to Gaussians. These values then used to fit the peaks in overall histograms which combine all the single molecule trajectories of each RNA complex.

## RESULTS

### *In vitro* reconstitution of the U4/U6 di-snRNP

To characterize the step-wise assembly of the U4/U6 di-snRNP, we first used EMSA by incubating purified proteins of the U4/U6 di-snRNP (Snu13, Prp31, Sm proteins, LSm proteins and Prp3/4, Figure 1B) with pre-formed U4/U6 snRNA duplex. The sequences, schematic representations of the constructs and the resulting data are shown in Supplementary Table S1, Supplementary Figure S1A, B and 2–17, respectively. The resulting apparent binding affinities (K_d, app_) are summarized in Table 1.

First, each U4/U6 protein or protein sub-complex was titrated against the preformed U4/U6 snRNA duplex. Snu13 binds to the duplex with higher affinity (K_d, app_ = 17 ± 1 nM, Supplementary Figure S2) indicating that the RNA is properly folded near the k-turn required for Snu13 binding. Previous EMSA studies have shown that Snu13 binding to U4 snRNA results in a shift at K_d, app_ = 75 nM Snu13, which is larger than the K_d, app_ obtained in our study (17). Prp3/4 binds the snRNA duplex with a K_d, app_ = 57 ± 2 nM (Supplementary Figure S3), in contrast with previous co-immunoprecipitation studies suggesting that human Prp3/4 requires the human Snu13 ortholog to bind U4/U6 snRNA duplex (8). The Sm protein subcomplex exhibits a K_d, app_ = 89 ± 4 nM (Supplementary Figure S4). Lastly, Prp31 alone binds the duplex with the lowest affinity (K_d, app_ = 243 ± 16 nM, Supplementary Figure S5), as expected given its known dependence upon a pre-formed k-turn RNA/Snu13 composite interface for high affinity binding (8).

To improve LSm homogeneity, we co-expressed the LSm components in yeast with multiple affinity tags (His-tag on LSm5 and CBP-tag on LSm8). This LSm complex exhibits a substantial improvement in RNP formation (44,45), particularly with truncated RNAs containing Snu13 and Prp31 (H46 RNA, Supplementary Figures S1B and S6). The improved LSm2–LSm8 complex binds to the full-length U4/U6 snRNA duplex with K_d, app_ = 5.0 ± 0.2 nM (Supplementary Figure S7) was obtained.

Then, we determined the binding affinity of each protein (Prp31, Sm, LSm and Prp3/4) with a fully formed U4/U6 snRNA/Snu13 ternary complex because Snu13 exhibits the smallest mobility shift due to its small size. As expected, the binding affinity of Prp31 was greatly increased in the presence of Snu13 (K_d, app_ = 50 ± 4 nM, Supplementary Figure S8). Sm proteins bound with no enhancement (K_d, app_ = 92 ± 9 nM, Supplementary Figure S9) indicating the lack of direct interactions between the Sm protein complex and Snu13 or the k-turn. LSm binding to the pre-formed U4/U6 snRNA/Snu13 complex was found to be lower (K_d, app_ = 26 ± 1 nM, Supplementary Figure S10) when compared to that observed for naked duplex RNA. The binding affinity of Prp3/4 for the pre-formed U4/U6 snRNA/Snu13 complex was only slightly higher (K_d,app_ = 88 ± 8 nM, Supplementary Figure S11).

For the next assembly stage, we determined the binding affinities of each of the remaining components (Sm, LSm and Prp3/4) under conditions ensuring complete formation of the U4/U6 snRNA/Snu13/Prp31 quaternary complex. Both the Sm protein and LSm protein binding affinities remain high (K_d, app_ = 108 ± 9 nM and 98 ± 34 nM respectively, Supplementary Figures S12 and S13). However, Prp3/4 exhibits significantly weaker binding to the pre-formed U4/U6 snRNA/Snu13/Prp31 complex (K_d, app_ = 417 ± 43 nM, Supplementary Figure S14). This may be due to the presence of alternative conformations of the 3′-end of U6 snRNA in the absence of LSm proteins, which could interfere with Prp3/4 binding.

Next, we began with the fully formed U4/U6 snRNA/Snu13/Prp31/Sm complex and monitored LSm and Prp3/4 binding. LSm proteins bind the complex with comparable affinity (K_d, app_ = 152 ± 15 nM, Supplementary Figure S15), whereas Prp3/4 binding remains weak (K_d, app_ = 557 ± 75 nM, Supplementary Figure S16) compared to the naked U4/U6 snRNA duplex, consistent with steric hindrance by the free U6 3′ end on Prp3/4 binding. Finally, we examined Prp3/4 binding to the pre-formed U4/U6 snRNA/Snu13/Prp31/Sm/LSm complex. Interestingly, when all other U4/U6 components are present, the Prp3/4 binding affinity increases dramatically (K_d, app_ = 20 ± 2 nM, Supplementary Figure S17). This result suggests direct contact between LSm proteins and Prp3/4. Overall assembly along the pathway described proceeds with high-affinity for each component and Figure 1B demonstrates full U4/U6 di-snRNP complex assembly. This study also provides a systematic approach for the stepwise assembly of U4/U6 di-snRNP, which can be helpful for other snRNP assembly studies.

### Stems I and II are coaxially stacked

**Figure 2.**
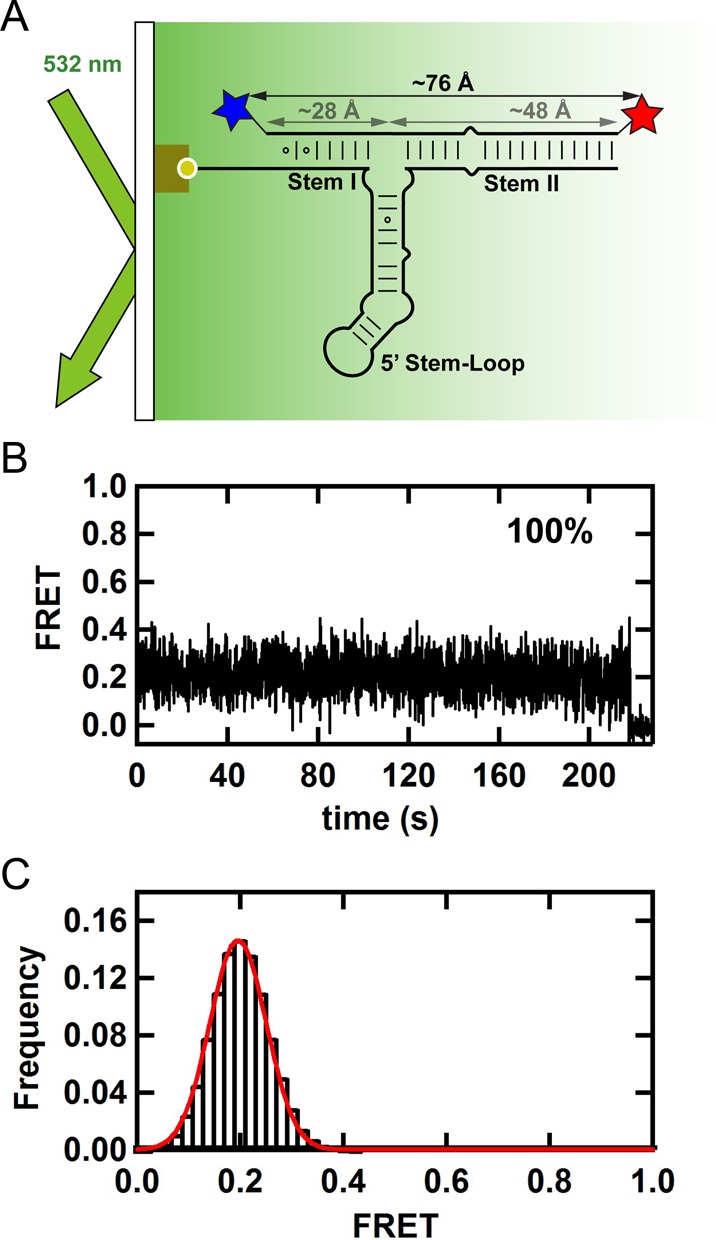
(**A**) Single-molecule FRET experimental setup with TIRF excitation. Surface immobilized 3′ end biotinylated U4 snRNA and Cy3-Cy5 labeled U6 snRNA at the 5′ and 3′ ends, respectively. (**B**) FRET time trajectory shows a single static FRET state and no dynamic transitions. (**C**) FRET histogram from 108 time-binned trajectories shows a peak at 0.2 FRET. The estimated distance for a 0.2 FRET value (76 Å) corresponds well to the sum total length of A-form stems I and II (28 and 48 Å, respectively), indicating that these stems are coaxially stacked in solution.

To characterize the global structure of the U4/U6 snRNA 3-way junction, we performed smFRET, as previously described (46,47). Minimal RNA constructs were designed, biotinylated, and fluorophore labeled (Supplementary Table S1 and Figure S1C) to monitor and to triangulate the relative position of each of the three helical arms (48,49). The minimal fluorophore labeled construct readily forms the whole RNP complex (Supplementary Figure S18), and protein binding does not affect the fluorescent properties of the dyes (Supplementary Figure S19). First, we looked at the relative orientation of stems I and II in the naked RNA duplex (Figure 2A). Examination of 108 single-molecule trajectories revealed a single static confirmation around 0.2 FRET (Figure 2B and C). The lack of structural dynamics indicates a rigid positioning of stems I and II. Using Forster's equation we estimated the distance between the two fluorophores to be ∼76 Å (R_0_ = 60 Å) (50). Considering that stem I is ∼10 base pairs long (∼28 Å assuming an A-form helix) and that stem II is ∼17 bp long (∼48 Å assuming an A-form helix), the experimentally estimated distance corresponds well to the sum total of these lengths (76 Å) strongly suggesting that stems I and II are coaxially stacked in solution (Figure 2A).

### The U4/U6 3-way junction is static

**Figure 3.**
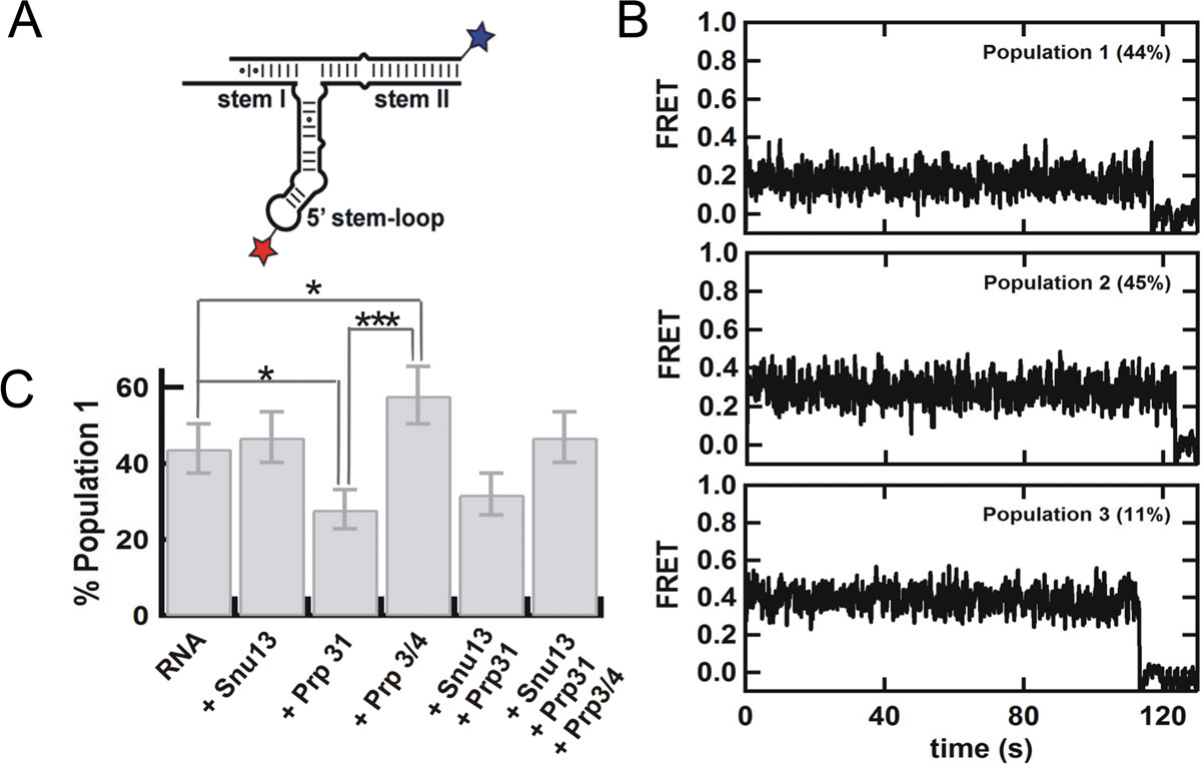
(**A**) Labeled construct to determine the orientation of stem II relative to the 5′ stem-loop by single-molecule FRET in the presence of the snRNP proteins (**B**) Representative single molecule trajectories for the three distinct populations observed by analysis of 102 trajectories in absence of proteins. (**C**) Changes in the fraction of population 1 in the presence of snRNP proteins in detriment of population 2. The fraction of population 3 remains constant under all conditions. *P* values are calculated using t-test and represent as * −*P* < 0.05, ** −*P* < 0.01 and *** −*P* < 0.001.

Next, we examined the orientation of the 5′ stem-loop relative to stem II in the naked snRNA duplex (Figure 3A). Analysis of 102 trajectories revealed the presence of three static and non-interconverting populations at 0.2 (44 ± 7%), 0.3 (45 ± 7%) and 0.4 (11 ± 3%) FRET (Figure 3B). This result indicates that the 5′ stem-loop can adopt multiple orientations relative to the statically stacked stems I and II, but each orientation is static and non-interconverting over the time scale of the experiment (minutes).

**Figure 4.**
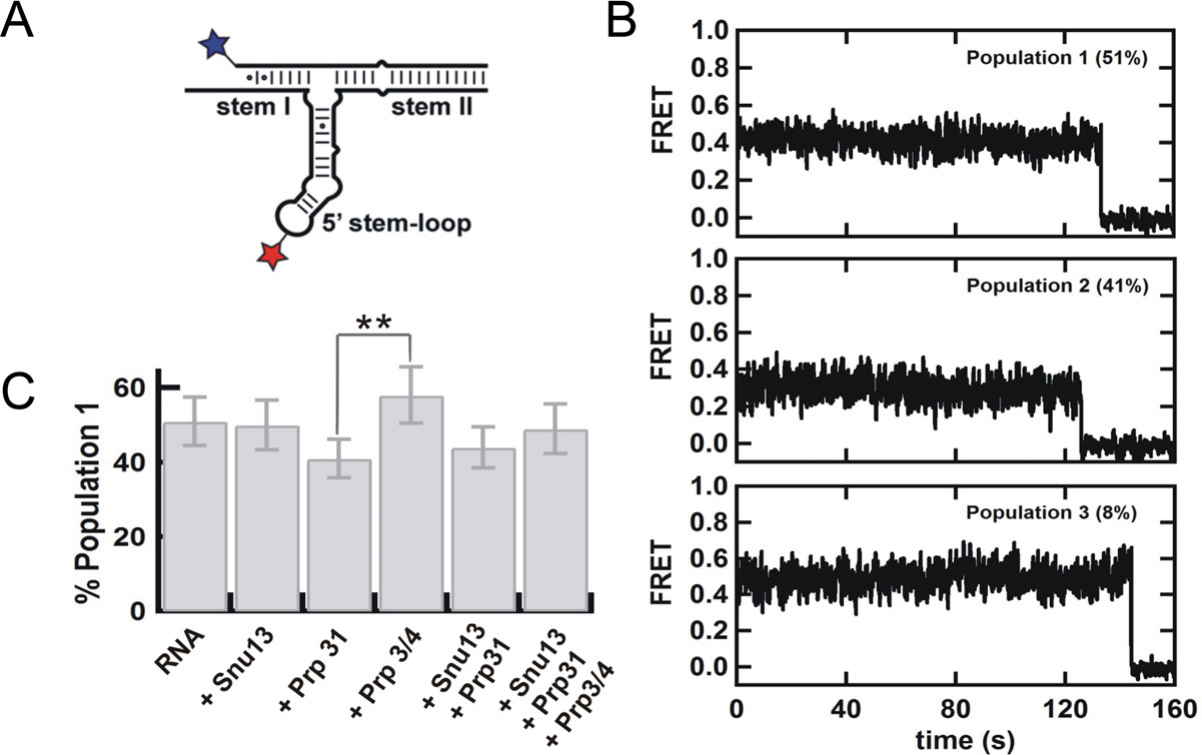
(**A**) Labeled construct to determine the orientation of stem I relative to the 5′ stem-loop by single-molecule FRET in the presence of the snRNP proteins (**B**) Representative single molecule trajectories for the three distinct populations observed by analysis of 105 trajectories in absence of proteins. (**C**) Changes in the fraction of population 1 in the presence of snRNP proteins in detriment of population 2. The fraction of population 3 remains constant under all conditions. *P* values are calculated using t-test and represent as * −*P* < 0.05, ** −*P* < 0.01 and *** −*P* < 0.001.

To corroborate this result, we examined the orientation of the 5′ stem-loop relative to stem I by labeling the opposite end of U6 snRNA (Figure 4A). Analysis of 105 trajectories also reveals the presence of three static populations (Figure 4B) with FRET values of 0.4 (51 ± 6%), 0.3 (41 ± 6%), and 0.5 (8 ± 3%) confirming the static heterogeneity observed in Figure 3B. Based on the FRET values and the relative populations in both experiments, we assign population 1 to a conformation with the 5′ stem loop closer to stem I, population 2 to a conformation with the 5′ stem-loop closer to stem II, and population 3 to a minor (possibly misfolded) population that is always present and that may correspond to a small population migrating between U6 and U4/U6 snRNA duplex as observed in the fluorescent EMSA (Supplementary Figure S18). This observation stands in marked contrast to the dynamic behavior found in other RNA junctions (46,48,49,51,52). A possible explanation for these conformations may be the existence of different orientations of the k-turn motif in the 5′ stem-loop (53–55), or different orientations of the three-way junction, as observed for other three-way junctions (49,51,56).

### The k-turn is preformed to facilitate Snu13 binding

To test whether these conformations result from different orientations of the k-turn motif, we introduced Snu13, which is expected to stabilize a single conformation upon binding (57,58). Analysis of 212 single-molecule trajectories under saturating concentrations of Snu13 showed no detectable change in the relative population fraction of the observed FRET conformations relative to either stem II (Figure 3C, Supplementary Figure S20) or stem I (Figure 4C) compared to the naked RNA. In contrast, the L7Ae protein, an archaeal Snu13 homolog (54,59), has been previously shown to stabilize a single conformation of a minimal k-turn construct (57). This result strongly indicates that the observed populations do not correspond to multiple orientations of the k-turn motif in the 5′ stem-loop. As expected, addition of Snu13 does not affect the relative orientation of stems I and II, which remain statically stacked in the presence of this protein (Supplementary Figure S21).

Increasing the concentration of Magnesium ions up to 50 mM in 100 mM NaCl, also results in no observable FRET changes or relative populations (Supplementary Figure S22). The lack of dynamics, FRET or population changes in the presence of Snu13 or Magnesium ions raises the interesting possibility that the U4/U6 k-turn is preformed to accelerate Snu13 binding and to promote further snRNP assembly. Here, the presence of high monovalent concentrations (100 mM NaCl) may help pre-fold the k-turn, as previously shown for the Kt-7 k-turn in the absence of protein (57).

### Prp31 preferentially binds one of two internal stem-loop conformations

To test whether these conformations result from different orientations of the three-way junction, we used Prp31, which interacts with the 5′ stem-loop and stem II (Figure 1A), thereby possibly affecting the three-way junction orientation relatively to the statically stacked stems I and II. We first looked at the orientation of the 5′ stem-loop relative to stem II (Figure 3A). Analysis of 107 trajectories in the presence of Prp31 shows a significant (*P* < 0.05) decrease in population 1 (from 44 ± 7% to 28 ± 5%, Figure 3C). This result indicates that Prp31 binding stabilizes the three-way junction in a conformation in which the 5′ stem-loop is farther from stem I and closer to stem II. To confirm this observation we look at the orientation of the 5′ stem-loop relative to stem I (Figure 4A). Consistent with this, the presence of Prp31 slightly decreases the fraction of population 1 (Figure 4C). The population changes in both experiments are in qualitative agreement and in the same direction, but interpreting three-dimensional changes in two dimensions may result in small assignment errors (∼few%) that account for the small differences between the two data sets. The presence of Prp31 does not affect the relative orientation of stems I and II, further supporting the idea that these two helices are rigidly and coaxially stacked even in the presence and absence snRNP proteins (Supplementary Figure S21). These data show that Prp31 preferentially binds and stabilizes a conformation of the U4/U6 snRNA 3-way junction that brings the 5′ stem-loop closer to stem II and further from stem I.

### Prp3/4 preferentially binds the alternative internal stem-loop conformation

We then looked at the effect of Prp3/4 binding on the orientation of the three-way junction. Prp3/4 binds to stem II near the three-way junction (Figure 1A), which could also result in changes to its conformation. First we looked at the orientation of the 5′ stem-loop relative to stem II (Figure 3A). Contrary to Prp31 binding, analysis of 105 single molecule trajectories shows a significant (*P* < 0.05) increase in population 1 (from 44 ± 7% to 58 ± 7%, Figure 3C), indicating that Prp3/4 binding stabilizes the conformation with the 5′ stem-loop closer to stem I. We then examined the orientation of the 5′ stem-loop relative to stem I (Figure 4A), which also revealed an increase in population 1, confirming this observation (Figure 4C). The relative orientation of stems I and II remains unchanged in the presence of Prp3/4, confirming that these two helices are stably stacked in the presence of any of the snRNP proteins (Supplementary Figure S21).

These data indicate that Prp3/4 preferentially binds and stabilizes a conformation of the U4/U6 snRNA 3-way junction in which the 5′ stem-loop is further from stem II and closer to stem I. The observation that Prp31 and Prp3/4 appear to each preferentially bind to and stabilize two alternative orientations of the 5′ stem-loop relative to the coaxially stacked stems I and II explains the previous finding that Prp3/4 binding affinity is significantly reduced after Prp31 is bound (Table 1) as the conformation of the RNA that is stabilized by Prp31 is not the preferred conformation for the binding of Prp3/4.

### Single-molecule assembly of multiple proteins onto the U4/U6 3-way junction RNA

**Figure 5.**
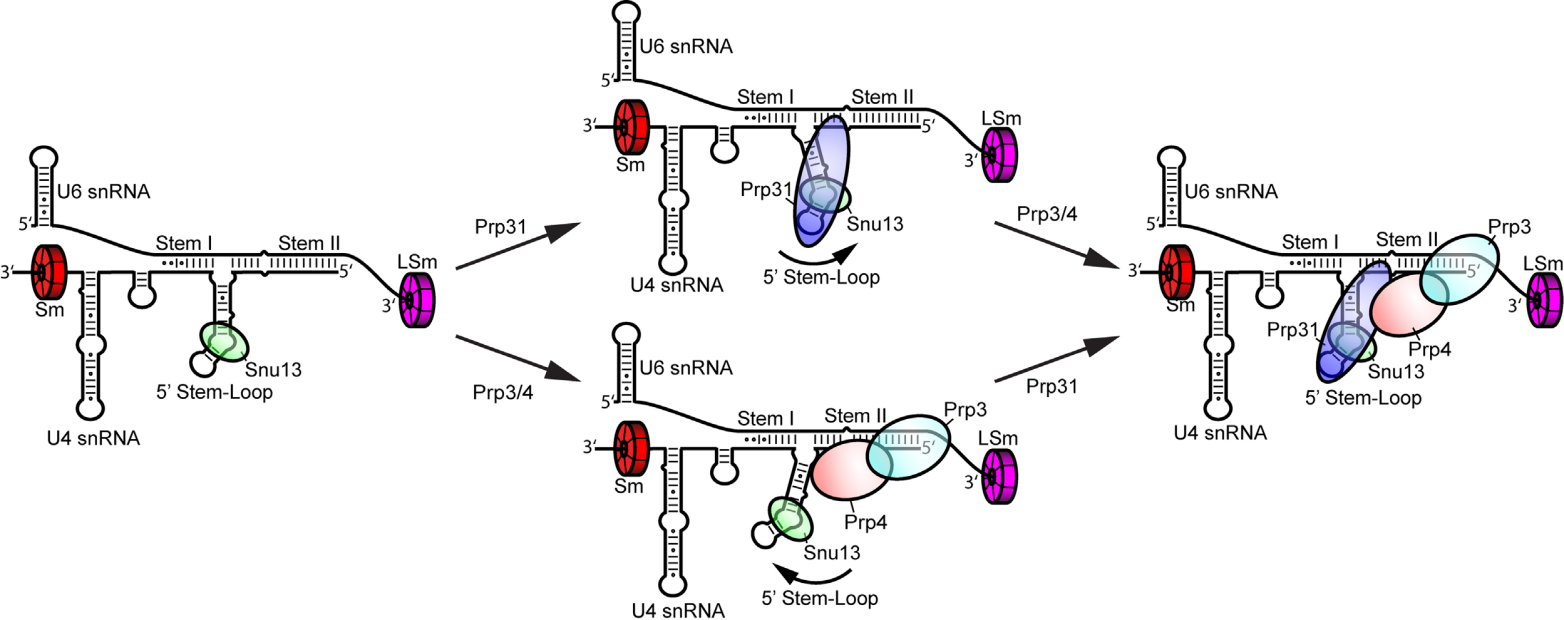
Assembly pathway of the U4/U6 di-snRNP *in vitro*. First, the Sm, LSm and Snu13 proteins bind independently and with high affinity to the U4 and U6 snRNAs. Subsequent assembly of the full complex may progress through binding of Prp31 followed by Prp3/4 (top pathway) or through Prp3/4 binding followed by Prp31 (bottom pathway). Binding of Prp31 stabilizes a conformation of the 5′ stem-loop closer to Stem II, whereas binding of Prp3/4 stabilizes a conformation of the 5′ stem-loop closer to Stem I. In the fully assembled complex the 5′ stem-loop returns to its initial orientation.

Next, we examined the conformational changes occurring under conditions where multiple proteins are bound to the RNA simultaneously. First, we looked at the relative orientation of stems I and II (Figure 2A). As expected from the individual protein experiments above, none of the snRNP proteins affect the observed FRET distributions, indicating that these two stems remain coaxially stacked during the entire snRNP assembly (Supplementary Figure S21, Figure 5) even on a time scale longer than 15 min (Supplementary Figure S23). Then, we checked the 5′ stem-loop orientation relative to stem II (Figure 3A). Analysis of 103 trajectories reveals that the presence of both Snu13 and Prp31 favors population 2 where Stem I is further from the 5′ stem-loop. In agreement with this, the presence of both Snu13 and Prp31 significantly decreases (*P* < 0.05) the fraction of population 1 to favor the conformation with the 5′ stem-loop closer to stem II (32 ± 6%, Figure 3C). A similar but smaller effect was observed with donor on stem I (Figure 4A and C). These data are consistent with the previous observation that the 5′ stem-loop moves toward stem II and away from stem I when Prp31 binds the U4/U6 snRNA 3-way junction.

Addition of Snu13, Prp31 and Prp3/4 to the RNA yields a population distribution that closely resembles that of the naked, or Snu13 bound RNA (Figures 3C and 4C), indicating that when both Prp31 and Prp3/4 are bound the relative stabilization of the alternative conformations observed in the intermediate assembly stages is lost. To confirm that Prp31 protein is bound to the U4/U6 snRNA duplex in the presence of Snu13 and Prp3/4, we performed single-molecule experiments using Cy5-labeled Prp31, biotinylated U4, Cy3-labeled U6 and alternated laser excitation between 532 nm and 637 nm (Supplementary Figure S24). In the presence of Cy5-Prp31, excitation at 532 nm results in a low FRET complex, while direct Cy5-excitation at 637 nm confirms that Cy5-Prp31 remains bound to the complex. These results confirm that Prp31 remains bound to the U4/U6 snRNA duplex even in the presence of Prp3/4 and Snu13.

## DISCUSSION

We have demonstrated that the U4/U6 di-snRNP can be assembled efficiently and with high affinities for all components under substoichiometric conditions (Figure 1B). Based on these results, we propose that Sm and LSm proteins pre-bind the U4 and U6 snRNAs, respectively, before duplex formation (60). This is consistent with previous studies showing that U6 snRNA, which is transcribed by Pol III (61), is assembled with LSm proteins in the nucleus (10,12,62), where it remains (63–65). Conversely, U4 snRNA is exported to the cytoplasm, where Sm proteins assemble, and transported back to the nucleus, where presumably other proteins assemble (66–70).

Although human Prp31 was not observed to bind the U4/U6 snRNA duplex significantly (8), yeast Prp31 binds yeast U4/U6 snRNA duplex with a K_d,app_ ∼240 nM. A recent U4/U6.U5 tri-snRNP structure determined by cryoEM single particle analysis (71) reveals extensive contacts between Prp31 and the 5′-stem of U4 snRNA. Our result suggests that Prp31 binds to the 5′-stem even in the absence of Snu13 with modest affinity. As previously shown for human (8,19), yeast Snu13 facilitates Prp31 binding to the k-turn of U4 snRNA, but only minimally affects the binding of Sm proteins, LSm proteins or Prp3/4 to the RNA. Therefore, our data suggest that Snu13 is an appropriate starting point for *in vitro* assembly (Figure 5), in accordance with the notion that Snu13 acts as a nucleating factor (8).

Similar binding affinities for Prp31 and Prp3/4 in the presence of Snu13 suggests that binding of each of these proteins onto the U4/U6 snRNA duplex occurs independently from each other, in agreement with previous studies (8,72). Binding of Sm proteins is unaffected by further addition of Prp31 as the Sm proteins do not interact with any other U4/U6 di-snRNP proteins.

Prp3/4 binds to naked U4/U6 snRNA duplex moderately well, but binding becomes substantially weaker when any of the other components, except LSm proteins, are prebound. It is interesting to note that both the LSm proteins and Prp3/4 bind less well after addition of Prp31, even though Prp31 and the WD40 domain of Prp4 seems in close contact in the tri-snRNP structure (71). The binding of Prp3/4 is greatly increased in the presence of the LSm proteins, consistent with the observation that the LSm protein ring contacts the ferredoxin-like domain of Prp3 (71).

Single-molecule experiments were done with the U4/U6 snRNA duplex labeled at different helices to determine their relative orientation. Our single-molecule data show that the U4/U6 snRNA duplex adopts and maintains a rigid or static global structure throughout di-snRNP formation. Interestingly, the experimentally determined inter-fluorophore distance within stem I and II using our single-molecule data is identical to the calculated total length of the two helices (assuming ideal A-form helices), suggesting that these two helices are coaxially stacked, forming a family A three-way junction conformation (26).

Despite the static global conformation of the U4/U6 snRNA duplex, we observed some heterogeneity within the population distribution of FRET states, where the two constructs designed to study the orientation of the 5′ stem-loop relative to stems I and II adopt two major, non-interconverting conformations. The minor (∼10%) high FRET population we consistently observed likely corresponds to a misfolded conformation.

Our single-molecule data show that the population fractions for the two conformations do not change upon Snu13 binding. This result indicates that under the experimental conditions used, the k-turn in U4 snRNA is already folded into a compact conformation, in accordance with previous studies (18,57). Hence, Snu13 binding does not cause further folding of the k-turn, which suggests that the observed population heterogeneity is independent of k-turn dynamics.

Furthermore, Prp31 binding stabilizes one observed population, whereas Prp3/4 stabilizes the other, indicating that binding of these two proteins to the U4/U6 snRNA duplex results in conformational changes within the duplex. Prp31 was shown to interact with the 5′ stem-loop of U4 snRNA and Prp3/4 binds to stem II (8,19,73). Thus, we suggest that binding of Prp31 moves the 5′ stem-loop toward stem II, whereas Prp3/4 shifts the stem-loop slightly toward stem I (Figure 5). The fully assembled complex adopts a conformation similar to what we observed for the naked snRNA duplex.

Taken together, we propose that the binding of individual proteins or partial assembly of di-snRNP can cause some local structural rearrangement, mainly in the 5′ stem-loop of U4 snRNA, whereas the stems I and II orientations remain unchanged. Lastly, binding of one protein can cause changes to the 5′ stem-loop conformation in a way that facilitates the binding of the other protein. Otherwise, the fully assembled complex upholds a static global conformation.

Our single-molecule data show that stems I and II of the U4/U6 snRNA duplex maintain a rigid helical conformation throughout the di-snRNP assembly. It is interesting to note that stems I and II are also coaxially stacked in the U4/U6.U5 tri-snRNP structure (71). As shown in previous studies, Brr2 plays an important role in the unwinding of the U4/U6 snRNA duplex to allow U6 snRNA to pair with U2 snRNA to form the active catalytic RNA core (74–77). On the basis of *in vitro* experiments it has been suggested that Brr2 translocates along U4 snRNA (78,79) and the recent cryoEM structure of the U4/U6.U5 tri-snRNP (71) show that the single-stranded region of U4 snRNA between its 3′ stem-loop and the U4/U6 snRNA stem I is loaded into the Brr2 helicase active site ready for unwinding. Previous studies have also proposed that U4/U6 snRNA duplex associated proteins may play an important role in the stabilization/destabilization of this duplex (8).

Unlike other RNAs that undergo protein induced structural changes during assembly (28,80), the structure of the U4/U6 snRNA duplex remains largely unchanged upon addition of divalent ions or proteins. Based on the sequence analysis and survey of RNA structures, it was originally predicted that stem II and the 5′ stem-loop of U4 snRNA are co-axially stacked (73) but it is now shown that stems I and II are stacked and this structure is stable and unaffected by protein binding. It will be interesting to determine the structure of this RNA by crystallography or NMR to determine how this 3-way junction helical arrangement is stabilized.

## Supplementary Material

SUPPLEMENTARY DATA
