# Supplementary material for: Assembly and dynamics of the U4/U6 di-snRNP by single-molecule FRET
Source: Nucleic Acids Res. 2015 Oct 25;43(22):10963–74. doi: 10.1093/nar/gkv1011 (PMC4678811; doi:10.1093/nar/gkv1011)
Supplement: SUPPLEMENTARY DATA [file supp_43_22_10963__index.html]

Assembly and dynamics of the U4/U6 di-snRNP by single-molecule FRET — SUPPLEMENTARY DATA 

# Assembly and dynamics of the U4/U6 di-snRNP by single-molecule FRET

## SUPPLEMENTARY DATA

- SUPPLEMENTARY DATA
