# Supplementary material for: Assembly and dynamics of the U4/U6 di-snRNP by single-molecule FRET
Source: Nucleic Acids Res. 2015 Oct 25;43(22):10963–74. doi: 10.1093/nar/gkv1011 (PMC4678811; doi:10.1093/nar/gkv1011)
Supplement: SUPPLEMENTARY DATA [file supp_gkv1011_nar-01787-r-2015-File007.docx]

**Assembly and dynamics of the U4/U6 di-snRNP by single-molecule FRET**

John Hardin^1,2,3,§^, Chandani Warnasooriya^1,2,§^, Yasushi Kondo^3,§^, Kiyoshi Nagai^3,^* and David Rueda^1,2,^*

^1^Department of Medicine, Section of Virology, Imperial College London, London W12 0NN, UK

^2^Single Molecule Imaging Group, MRC Clinical Sciences Centre, Imperial College London, London W12 0NN, UK

^3^MRC Laboratory of Molecular Biology, Cambridge CB2 0QH, United Kingdom

* Corresponding author: david.rueda@imperial.ac.uk or kn@mrc-lmb.cam.ac.uk

| **Name** | **Modifications** | **Sequence (5’ - 3’)** |
| --- | --- | --- |
| U4-full length | None | 5’-AUC CUU AUG CAC GGG AAA UAC GCA UAU CAG UGA GGA UUC GUC CGA GAU UGU GUU UUU GCU GGU UGA AAU UUA AUU AUA AAC CAG ACC GUC UCC UCA UGG UCA AUU CGG UGU UCG CUU UUG AAU ACU UCA AGA CUA UGU AGG GAA UUU UUG GAA UAC CUU U-3’ |
| U6-full length | None | 5’-GUU CGC GAA GUA ACC CUU CGU GGA CAU UUG GUC AAU UUG AAA CAA UAC AGA GAU GAU CAG CAG UUC CCC UGC AUA AGG AUG AAC CGU UUU ACA AAG AGA UUU AUU UCG UUU U-3’ |
| H46 RNA | None | 5’-AUC CUU AUG CAC GGG AAA UAC GCA UAU CAG UGA GGA UUC GUC CGA GAU UGU GUU UUU GCU GGU UGA AAG AUC AGC AGU UCC CCU GCA UAA GGA UGA ACC GUU UUA CAA AGA GAU UUA UUU CGU UUU-3’ |
| U4 | X-(5-LC-NU)-amino modifier for Cy5 labeling | 5’-AUC CUU AUG CAC GGG AAA UAC GCA UAU CAG UGA GGA UXC GUC CGA GAU UGU GUU UUU GCU GGU UGA AAU UU-(3’-Biotin) |
| U6-I | None | 5’-Cy3-UGA UCA GCA GUU CCC CUG CAU AAG GAU |
| U6-II | Y- C6 amino modifier for Cy3 labeling | 5’-UGA UCA GCA GUU CCC CUG CAU AAG GAU Y |
| U6-III | Z- C6 amino modifier for Cy5 labeling | 5’-Cy3-UGA UCA GCA GUU CCC CUG CAU AAG GAU Z |

**Supplementary Table 1:** RNA sequences used in this study.


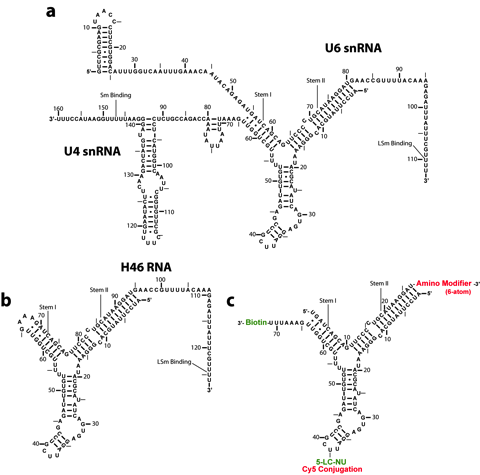


**Supplementary Figure 1:** RNA constructs used in this study. (a) Full length U4/U6 snRNA duplex. (b) H46 RNA. A truncated U4/U6 hybrid RNA construct generated by capping off stem I with a GA_3_ tetra loop. (c) A truncated U4/U6 snRNA duplex used in single-molecule studies, U4 contains a 5-LC-NU internal amino modifier for cy5 labeling and 3’ biotin. U6 is either labeled with Cy3 at the 5’ end (U6-I) and contains a C6 amino modifier for Cy5 labeling (U6-III) or contains a C6 amino modifier at the 3’ end for Cy3 labeling (U6-II).

**
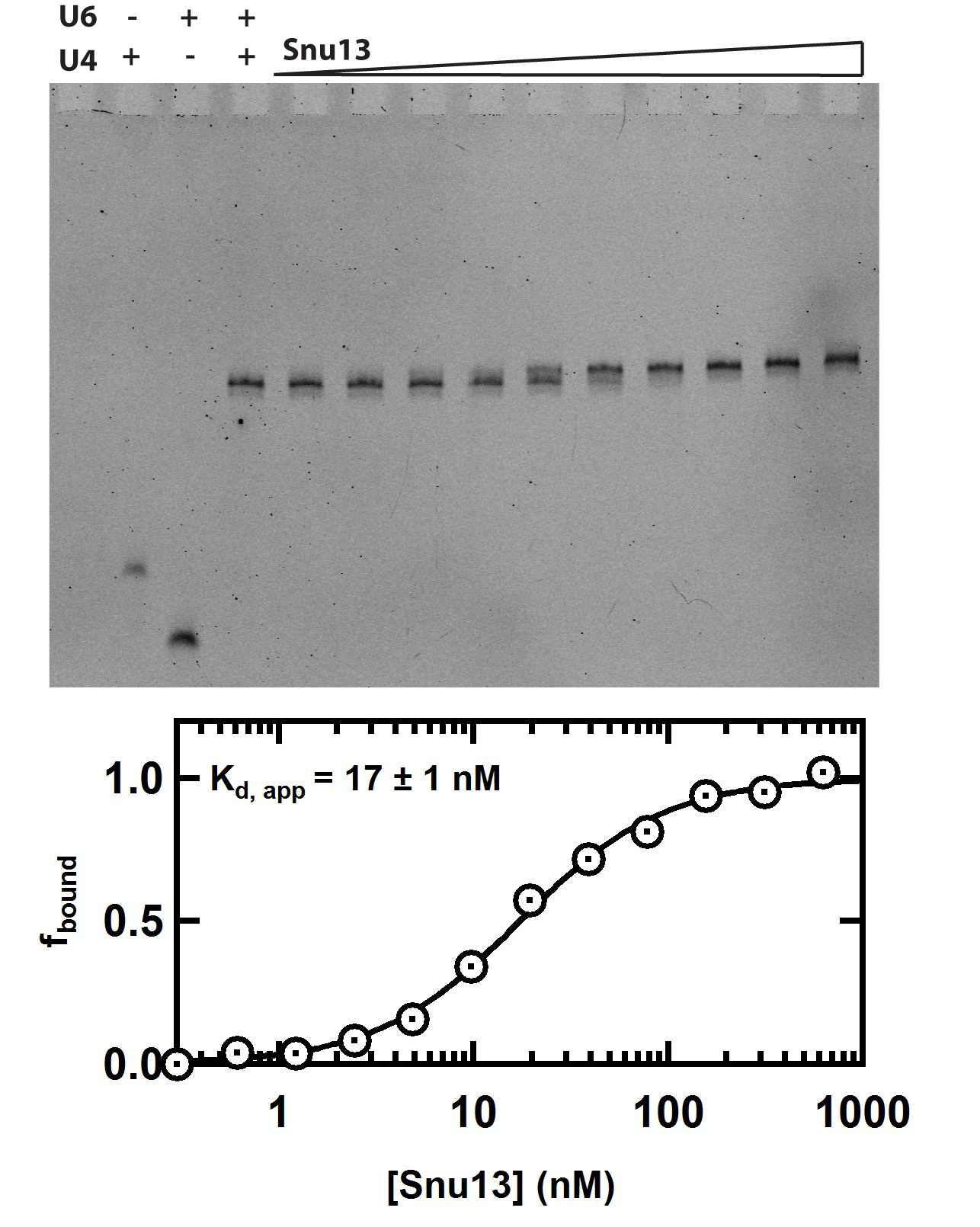
**

**Supplementary Figure 2:** Gel shift and binding curve for the interaction between the pre-formed U4/U6 snRNA duplex and Snu13. The concentration of pre-formed U4/U6 snRNA duplex is 2 nM. The concentration of Snu13 ranges from 0-5 μM in 2-fold increments. The apparent K_d_ (K_d, app_) and estimated Hill coefficient (n) is given for a single representative binding curve.

**
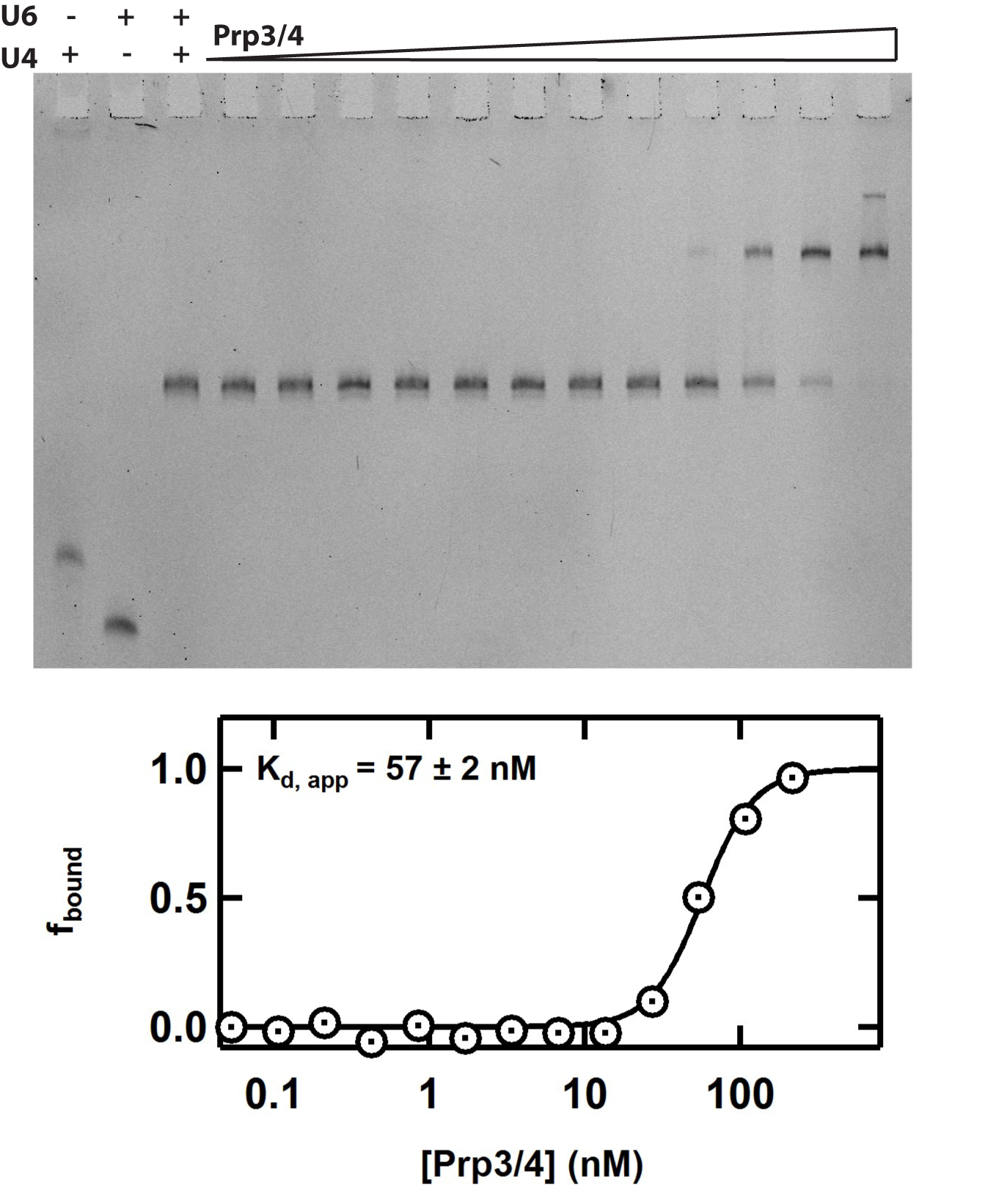
**

**Supplementary Figure 3:** Gel shift and binding curve for the interaction between the pre-formed U4/U6 snRNA duplex and Prp3/4. The concentration of pre-formed U4/U6 snRNA duplex is 2 nM. The concentration of Prp3/4 ranges from 0-3.47 μM in 2-fold increments. The apparent K_d_ (K_d, app_) and estimated Hill coefficient (n) is given for a single representative binding curve.

**
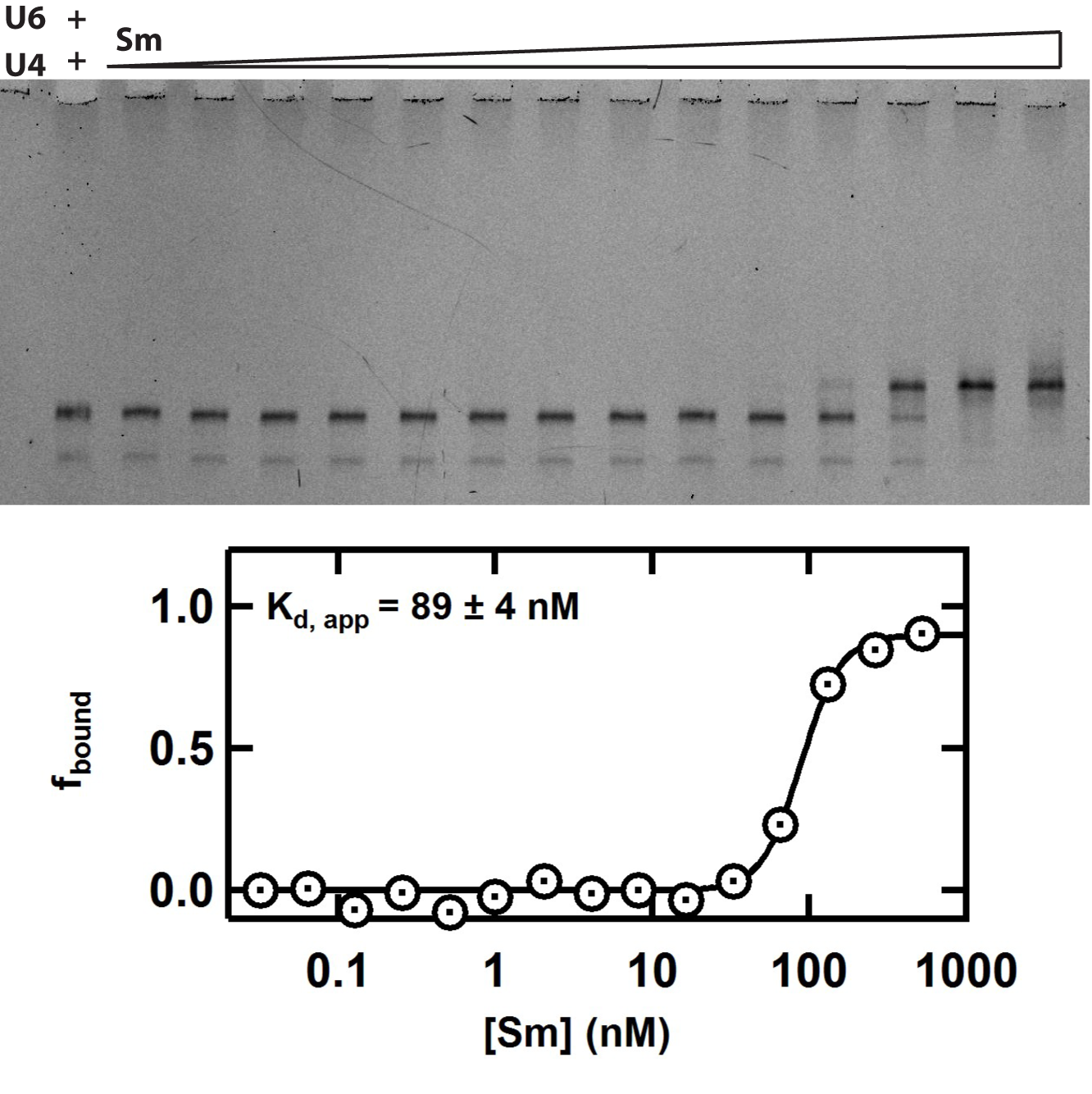
**

**Supplementary Figure 4:** Gel shift and binding curve for the interaction between the pre-formed U4/U6 snRNA duplex and Sm proteins. The concentration of pre-formed U4/U6 snRNA duplex is 2 nM. The concentration of Sm ranges from 0-2.1 μM in 2-fold increments. The apparent K_d_ (K_d, app_) and estimated Hill coefficient (n) is given for a single representative binding curve.

**
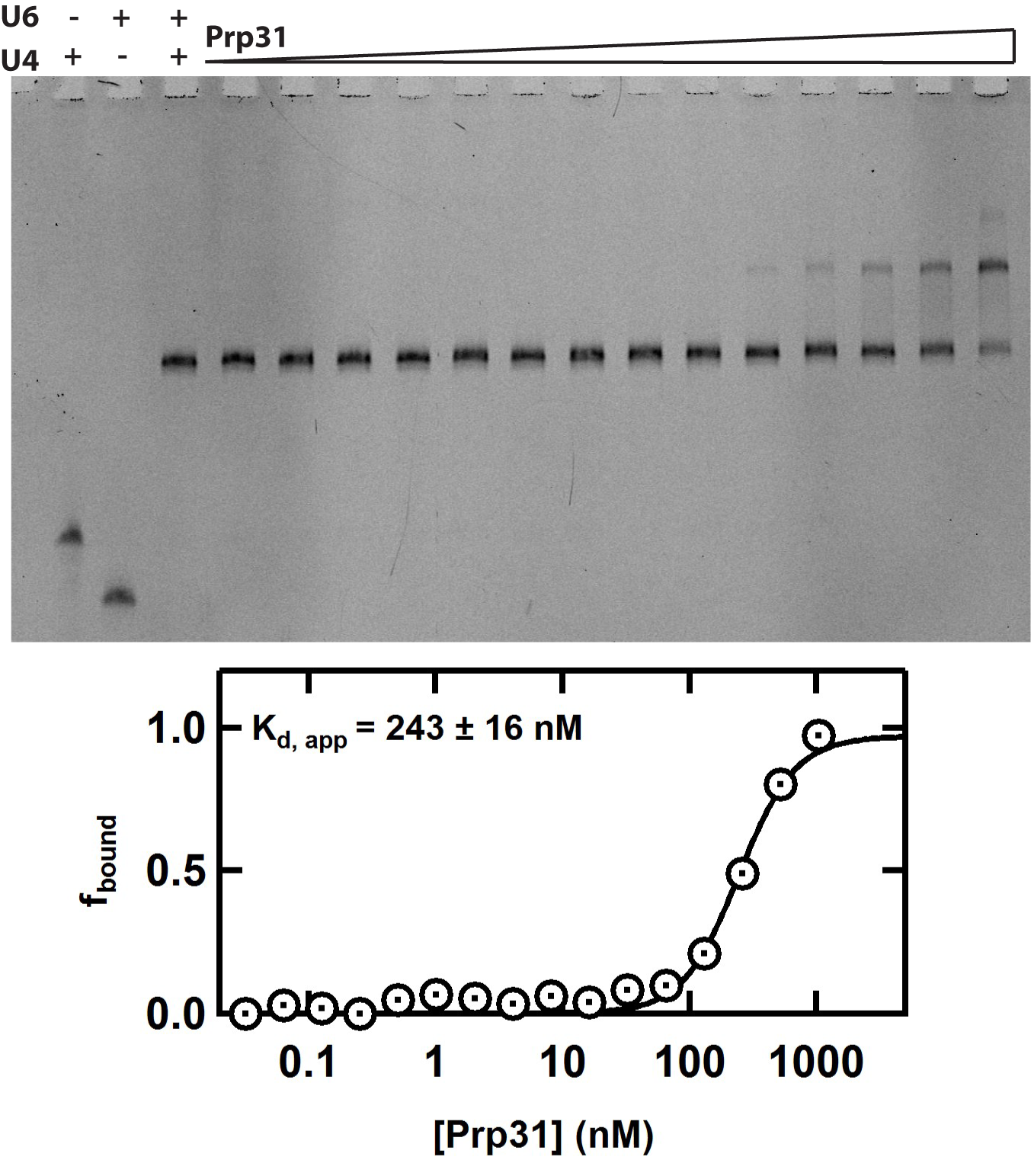
**

**Supplementary Figure 5:** Gel shift and binding curve for the interaction between the pre-formed U4/U6 snRNA duplex and Prp31. The concentration of pre-formed U4/U6 snRNA duplex is 2 nM. The concentration of Prp31 ranges from 0-2.08 μM in 2-fold increments. The apparent K_d_ (K_d, app_) and estimated Hill coefficient (n) is given for a single representative binding curve.


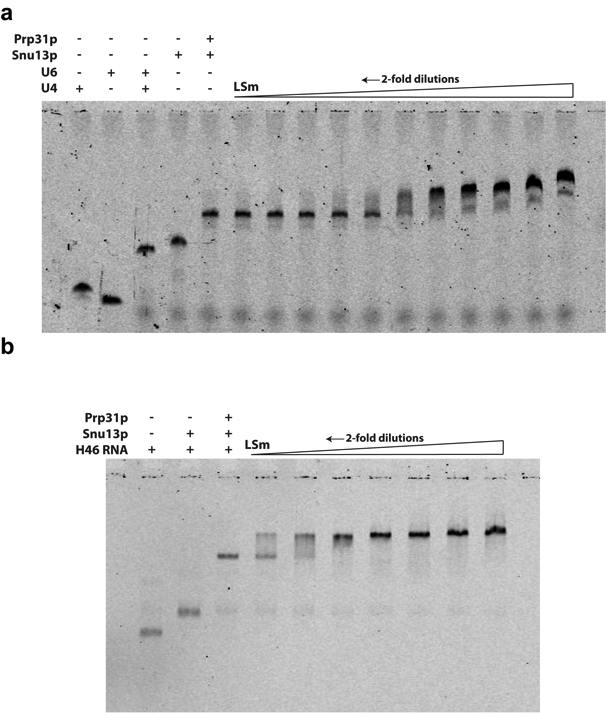


**Supplementary Figure 6:** Gel-shifts demonstrating behavior of LSm containing constructs with (a) Full length U4/U6 snRNA duplex and (b) the H46 snRNA. In both cases, Snu13 and Prp31 containing complexes were pre-formed before titration with the LSm complex.

**
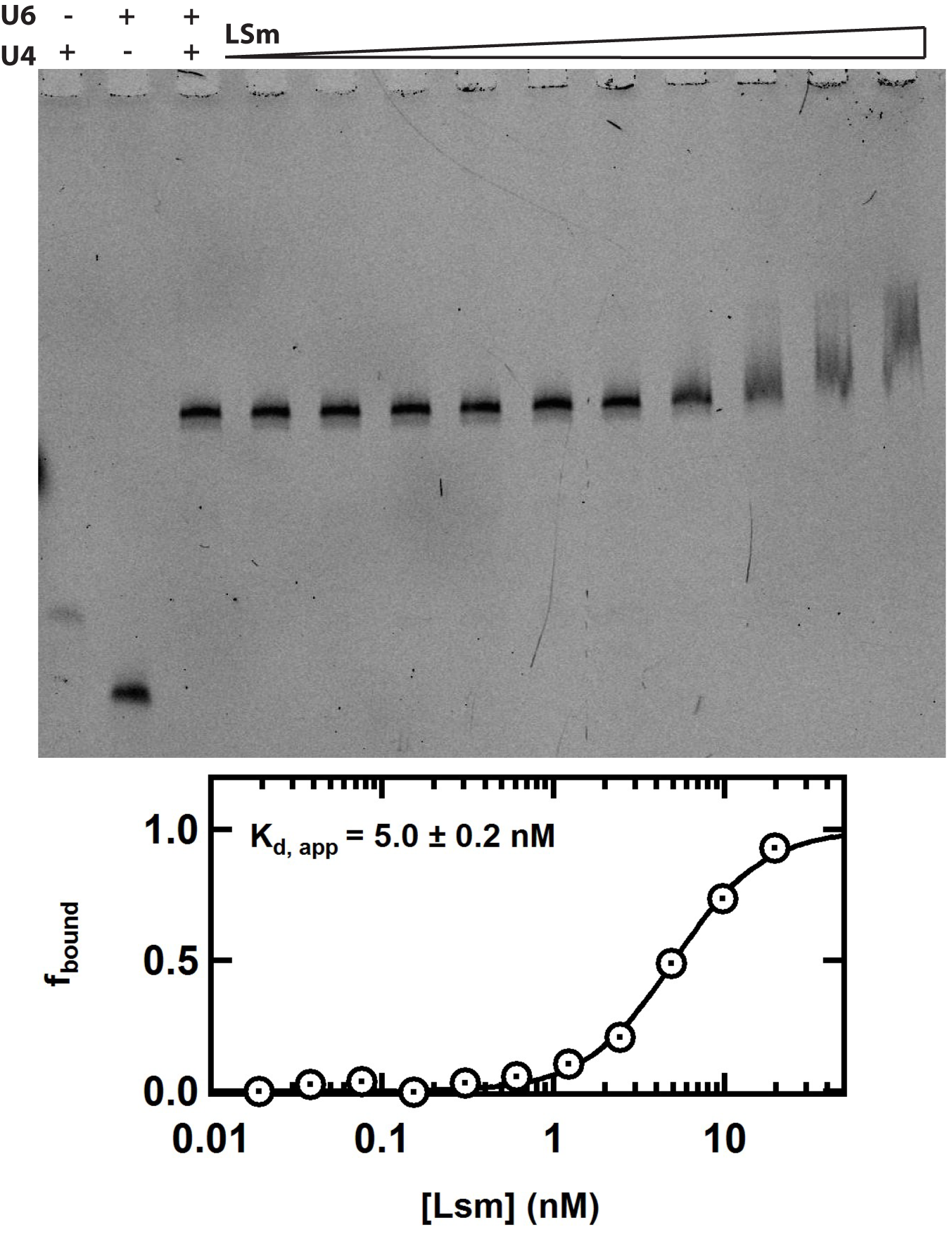
**

**Supplementary Figure 7:** Gel shift and binding curve for the interaction between the pre-formed U4/U6 snRNA duplex and LSm proteins. The concentration of pre-formed U4/U6 snRNA duplex is 2 nM. The concentration of LSm ranges from 0-1.25 μM in 2-fold increments. The apparent K_d_ (K_d, app_) is given for a single representative binding curve.

**
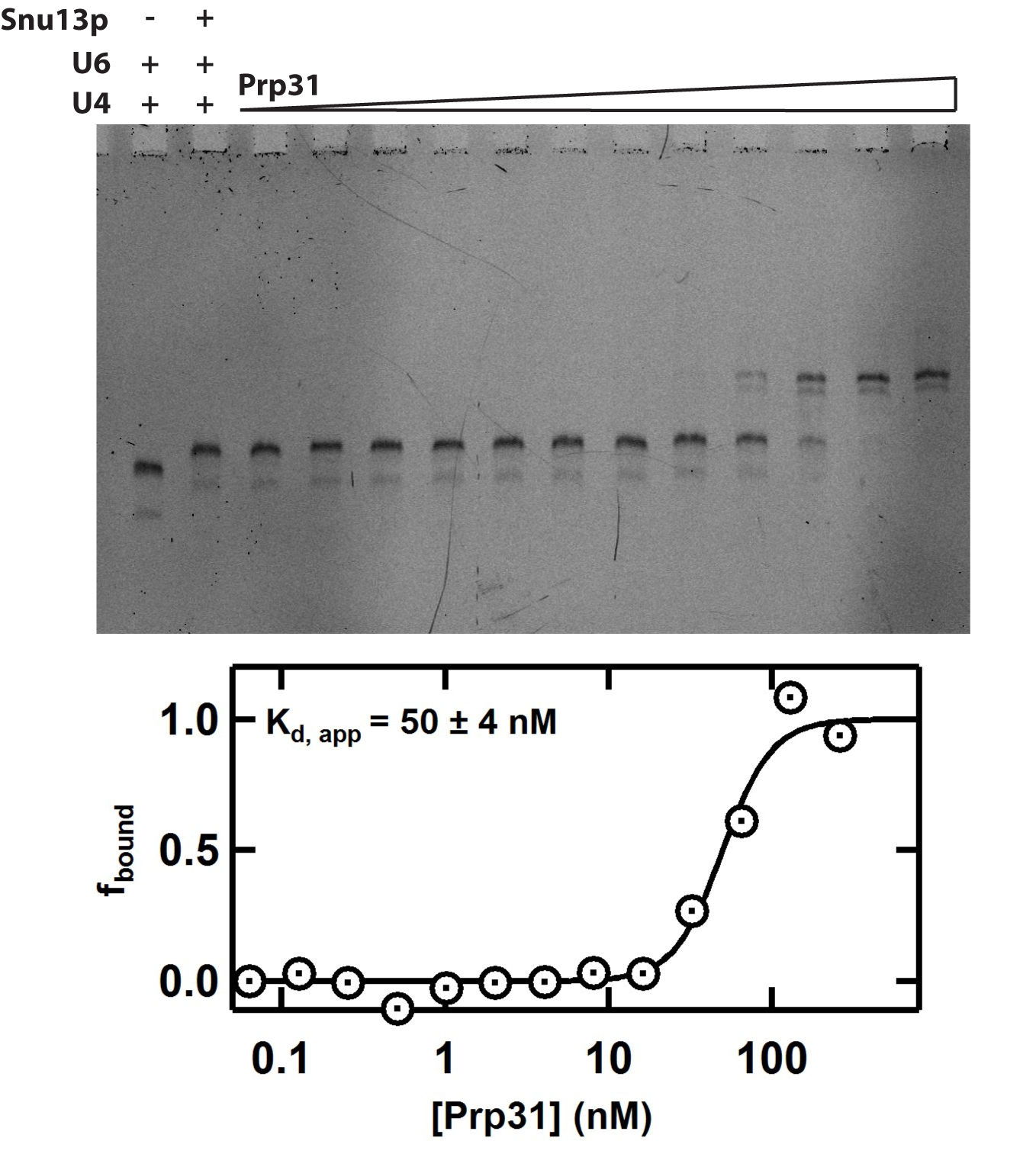
**

**Supplementary Figure 8:** Gel shift and binding curve for the interaction between the pre-formed U4/U6 snRNA/Snu13 complex and LSm proteins. The concentrations of pre-formed complex components are U4/U6 snRNA duplex (2 nM), and Snu13 (200 nM). The concentration of LSm ranges from 0-2.5 μM in 2-fold increments. The apparent K_d_ (K_d, app_) is given for a single representative binding curve.

**
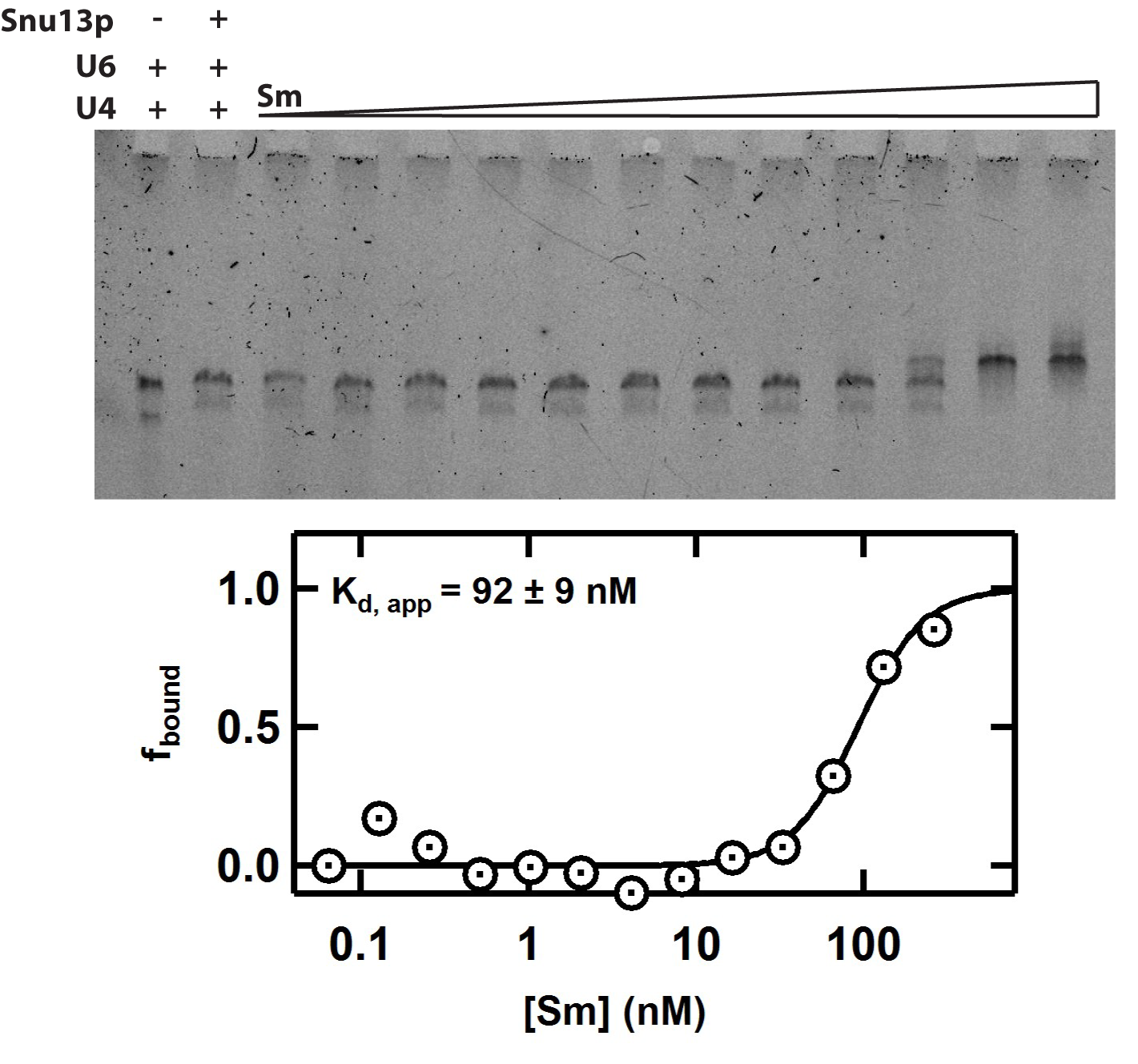
**

**Supplementary Figure 9:** Gel shift and binding curve for the interaction between the pre-formed U4/U6 snRNA/Snu13 complex and Sm proteins. The concentrations of pre-formed complex components are U4/U6 snRNA duplex (2 nM), and Snu13 (200 nM). The concentration of Sm ranges from 0-2.1 μM in 2-fold increments. The apparent K_d_ (K_d, app_) is given for a single representative binding curve.

**
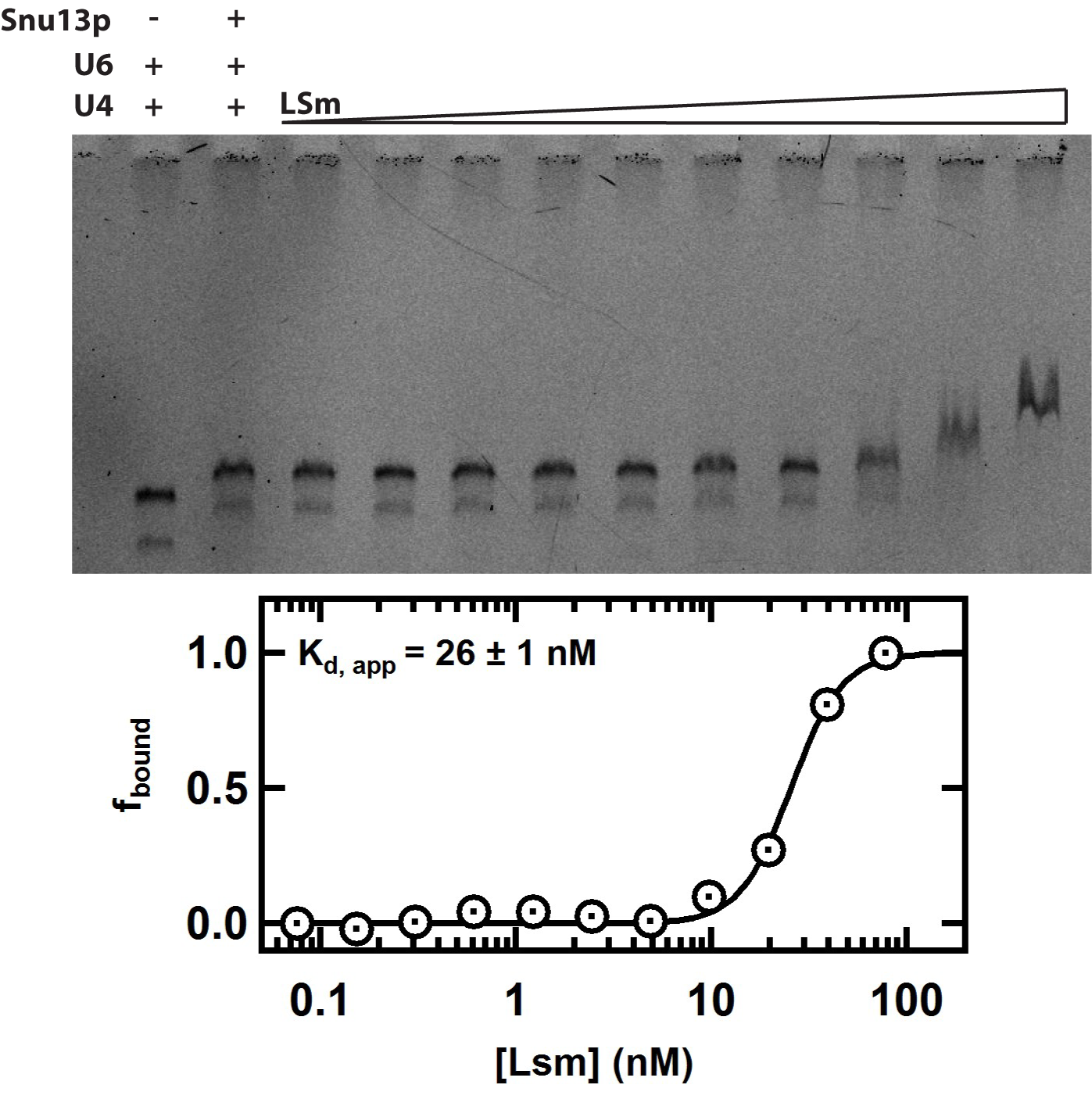
**

**Supplementary Figure 10:** Gel shift and binding curve for the interaction between the pre-formed U4/U6 snRNA/Snu13 complex and LSm proteins. The concentrations of pre-formed complex components are U4/U6 snRNA duplex (2 nM), and Snu13 (200 nM). The concentration of LSm ranges from 0-2.5 μM in 2-fold increments. The apparent K_d_ (K_d, app_) is given for a single representative binding curve.

**
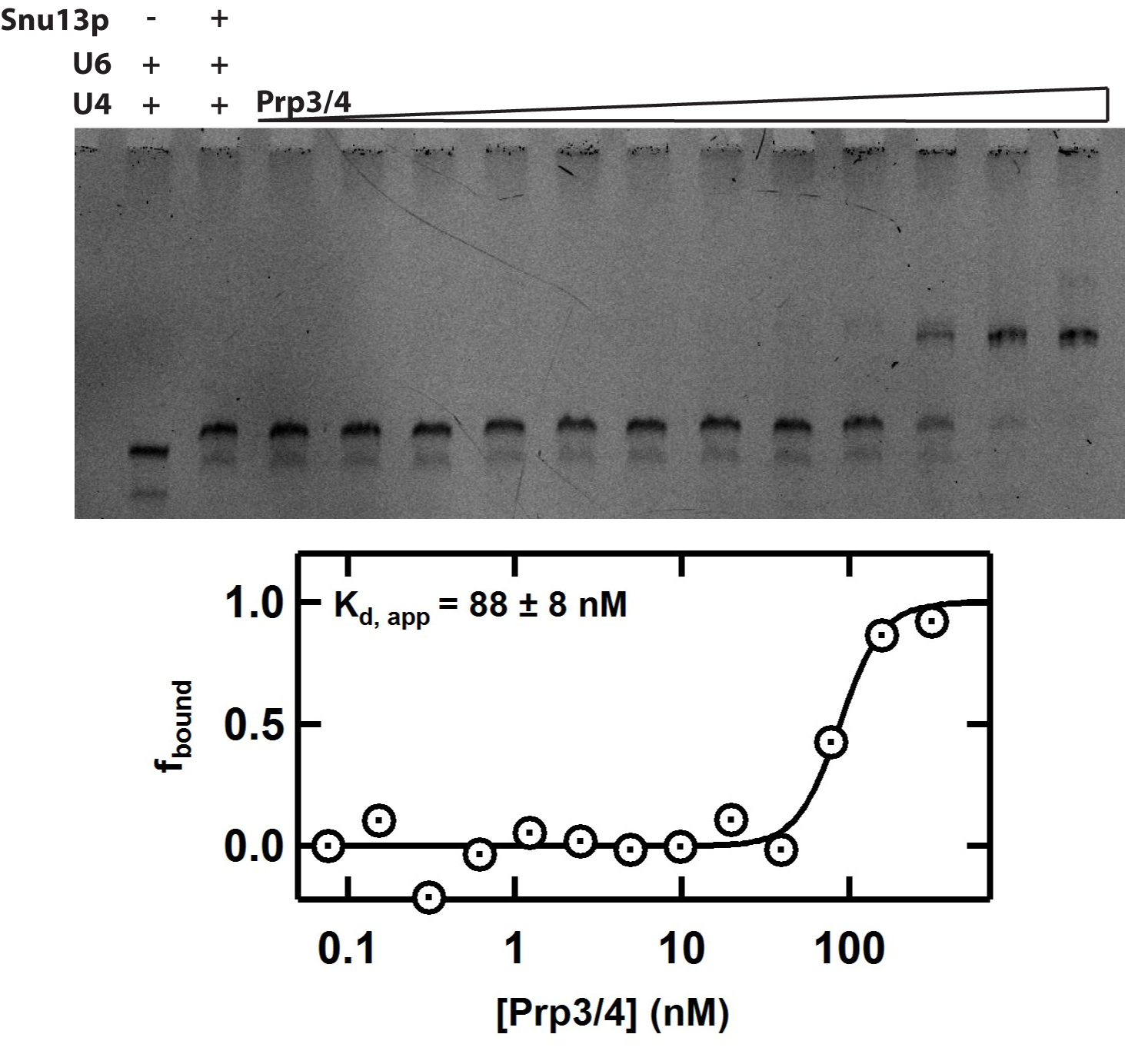
**

**Supplementary Figure 11:** Gel shift and binding curve for the interaction between the pre-formed U4/U6 snRNA/Snu13 complex and Prp3/4. The concentrations of pre-formed complex components are U4/U6 snRNA duplex (2 nM), and Snu13 (200 nM). The concentration of Prp3/4 ranges from 0-2.5 μM in 2-fold increments. The apparent K_d_ (K_d, app_) is given for a single representative binding curve.

**
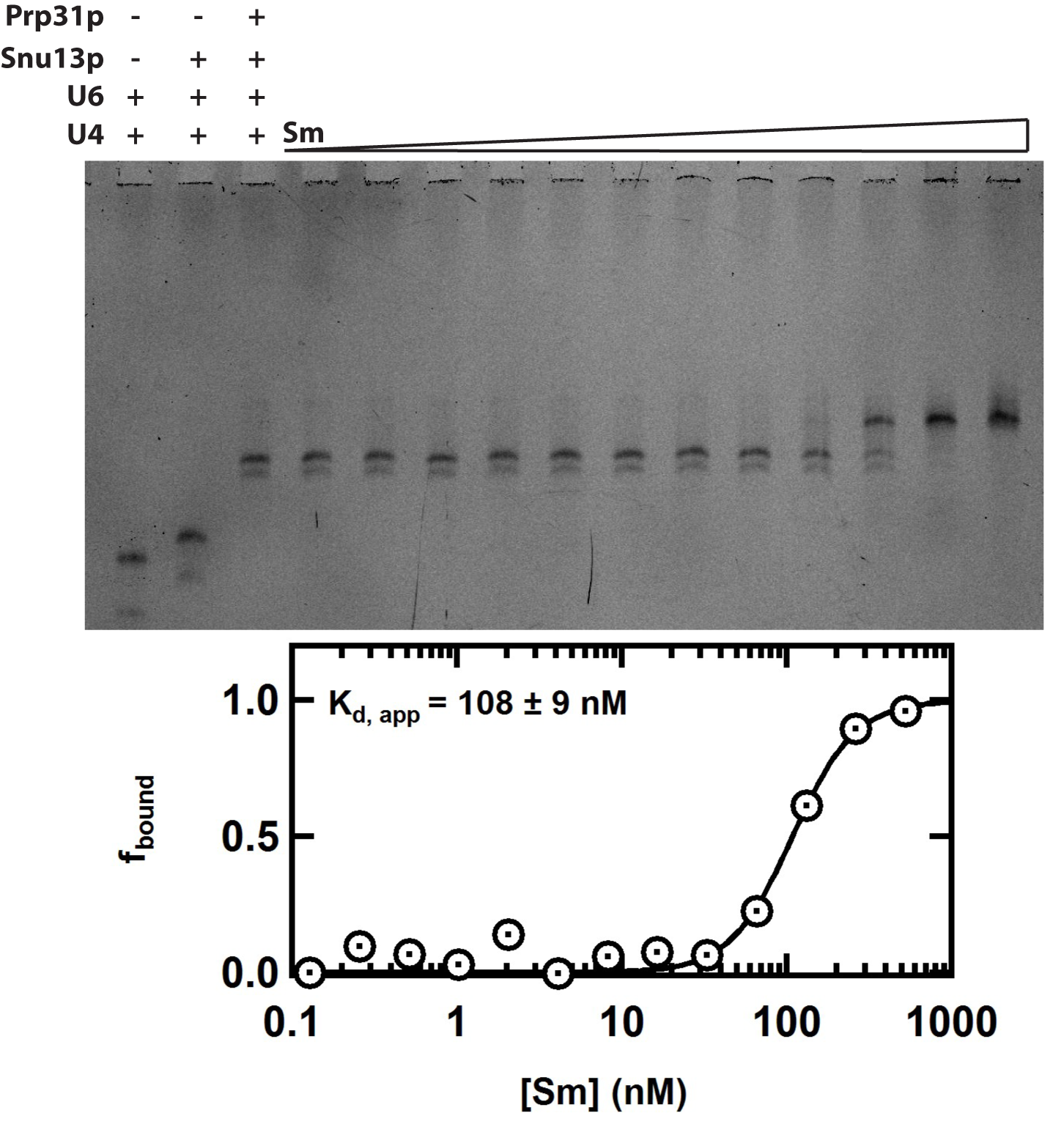
**

**Supplementary Figure 12:** Gel shift and binding curve for the interaction between the pre-formed U4/U6 snRNA/Snu13/Prp31 complex and Sm proteins. The concentrations of pre-formed complex components are U4/U6 snRNA duplex (2 nM), Snu13 (200 nM), and Prp31 (120 nM). The concentration of Sm ranges from 0-2.1 μM in 2-fold increments. The apparent K_d_ (K_d, app_) is given for a single representative binding curve.


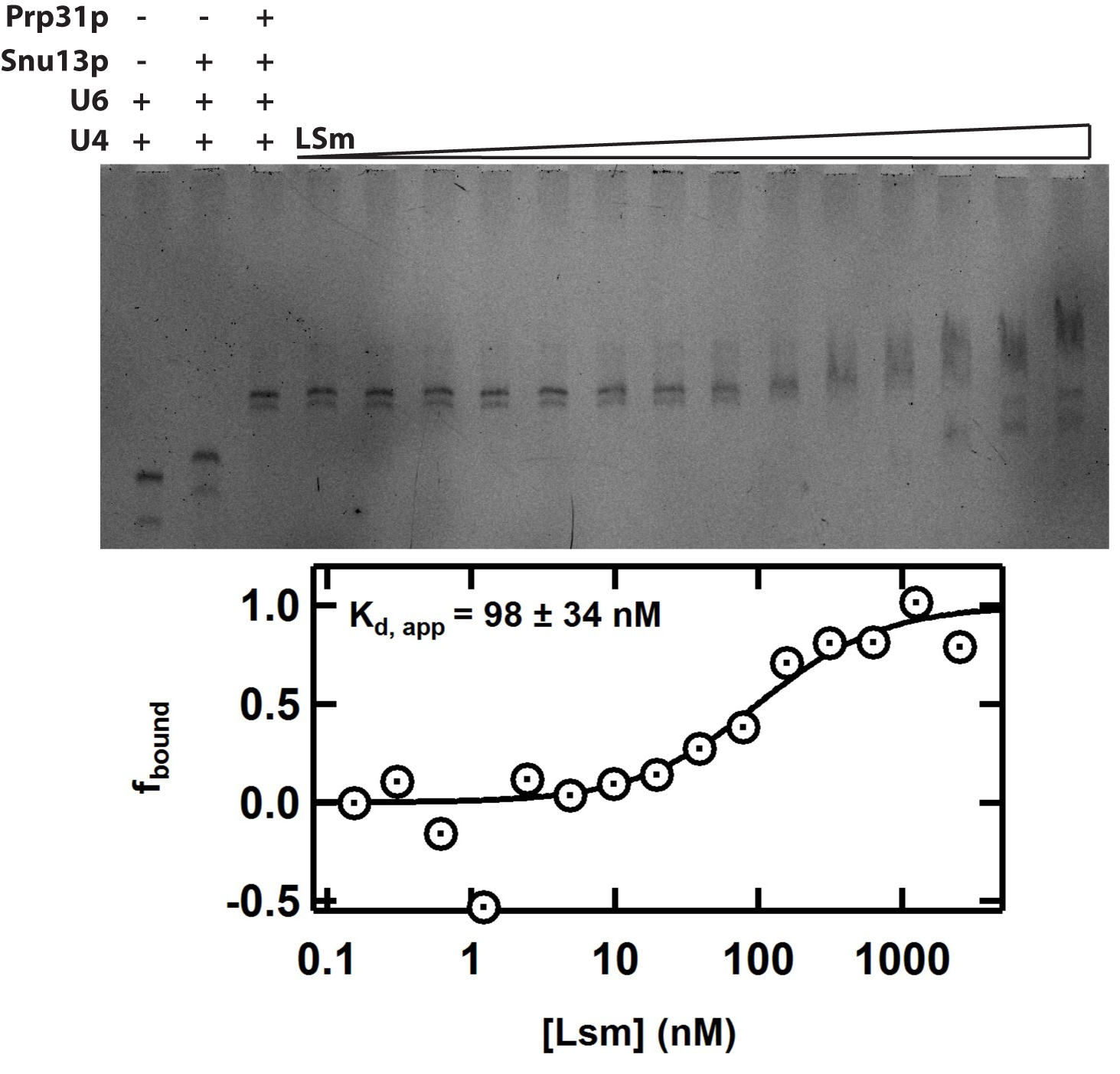


**Supplementary Figure 13:** Gel shift and binding curve for the interaction between the pre-formed U4/U6 snRNA/Snu13/Prp31 complex and LSm proteins. The concentrations of pre-formed complex components are U4/U6 snRNA duplex (2 nM), Snu13 (200 nM), and Prp31 (120 nM). The concentration of LSm ranges from 0-2.5 μM in 2-fold increments. The apparent K_d_ (K_d, app_) is given for a single representative binding curve.

**
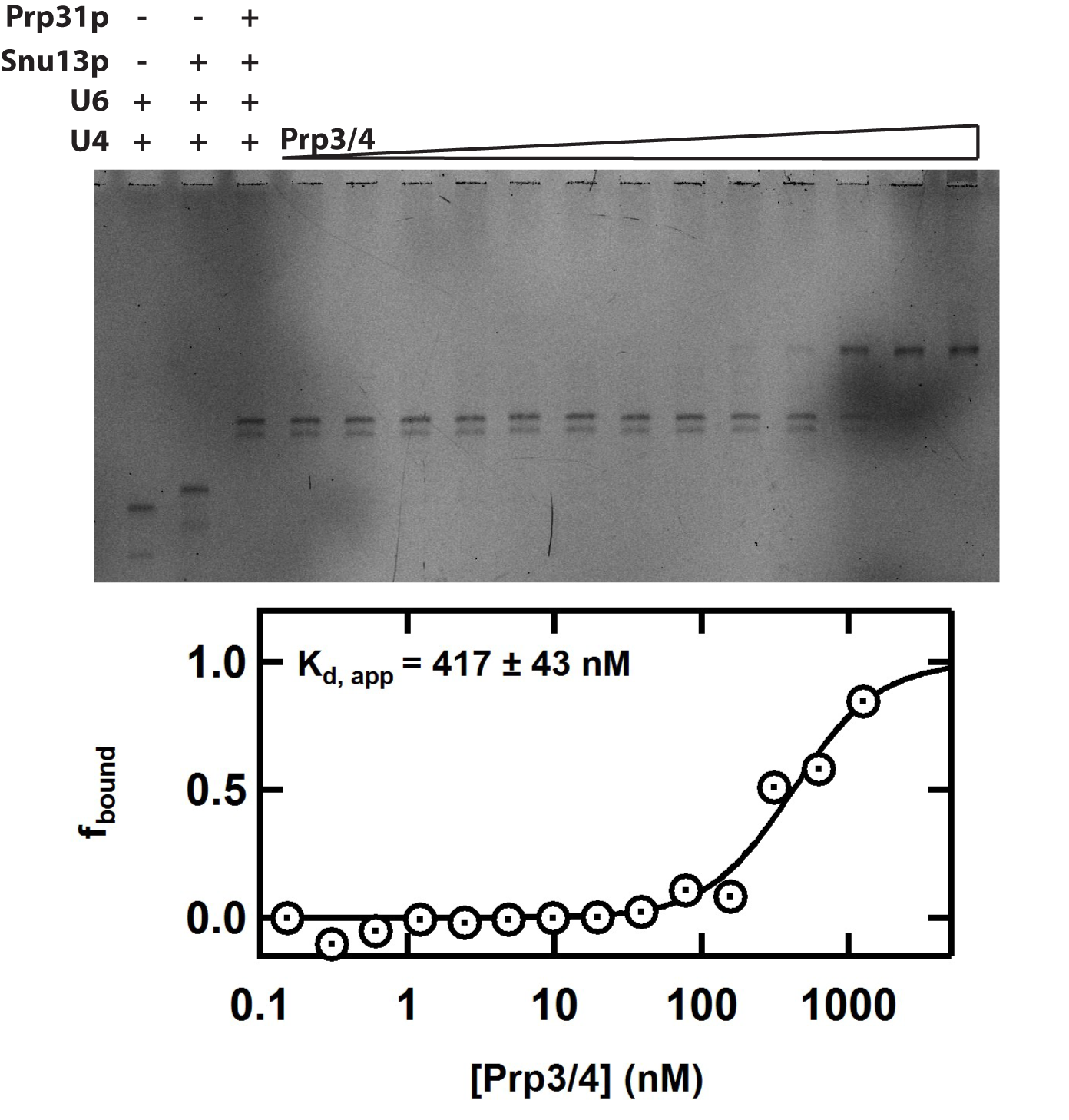
**

**Supplementary Figure 14:** Gel shift and binding curve for the interaction between the pre-formed U4/U6 snRNA/Snu13/Prp31 complex and Prp3/4. The concentrations of pre-formed complex components are U4/U6 snRNA duplex (2 nM), Snu13 (200 nM), and Prp31 (120 nM). The concentration of Prp3/4 ranges from 0-2.5 μM in 2-fold increments. The apparent K_d_ (K_d, app_) is given for a single representative binding curve.

**
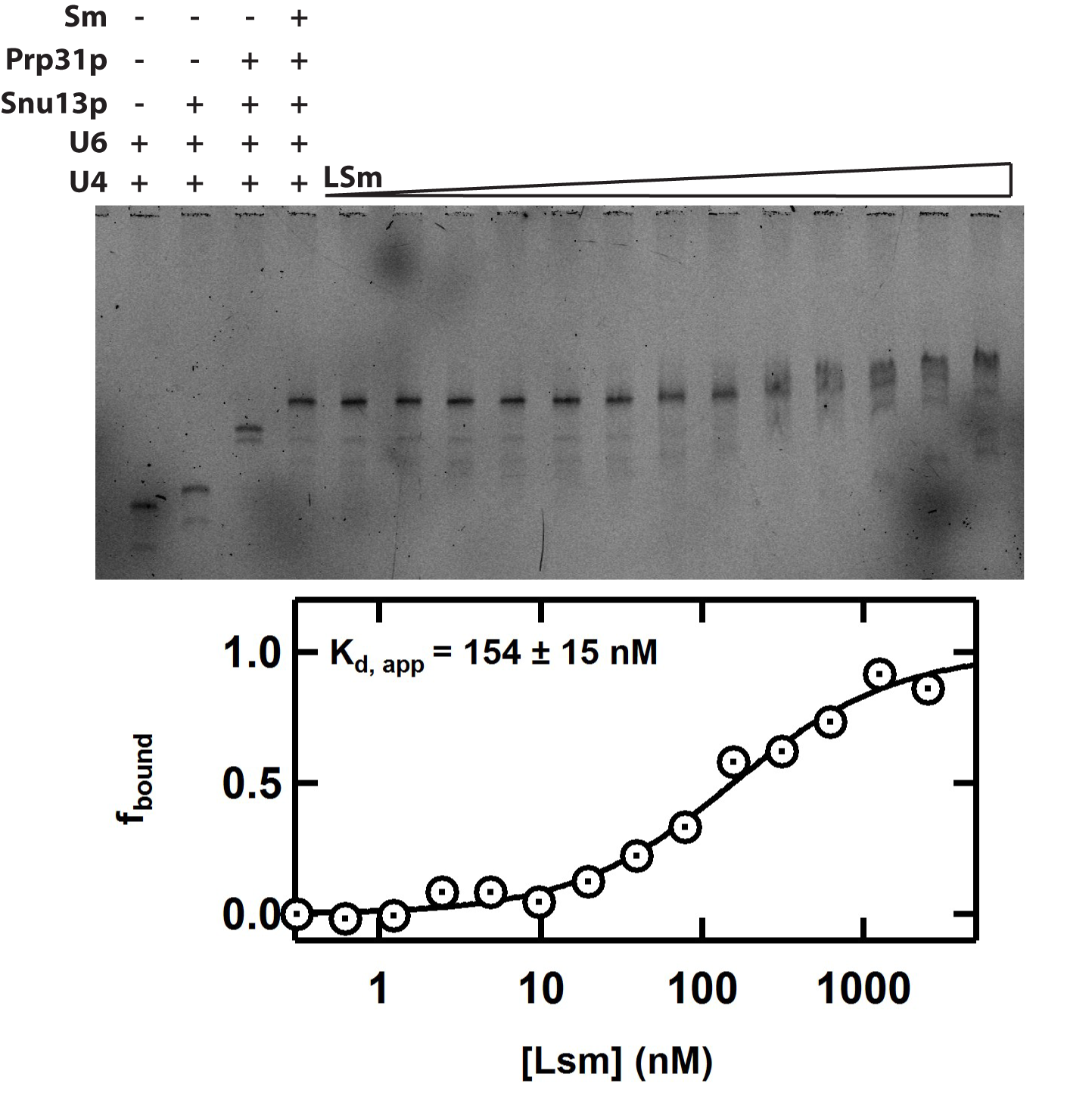
**

**Supplementary Figure 15:** Gel shift and binding curve for the interaction between the pre-formed U4/U6 snRNA/Snu13/Prp31/Sm complex and LSm proteins. The concentrations of pre-formed complex components are U4/U6 snRNA duplex (2 nM), Snu13 (200 nM), Prp31 (120 nM), and Sm proteins (64 nM). The concentration of LSm ranges from 0-2.5 μM in 2-fold increments. The apparent K_d_ (K_d, app_) is given for a single representative binding curve.

**
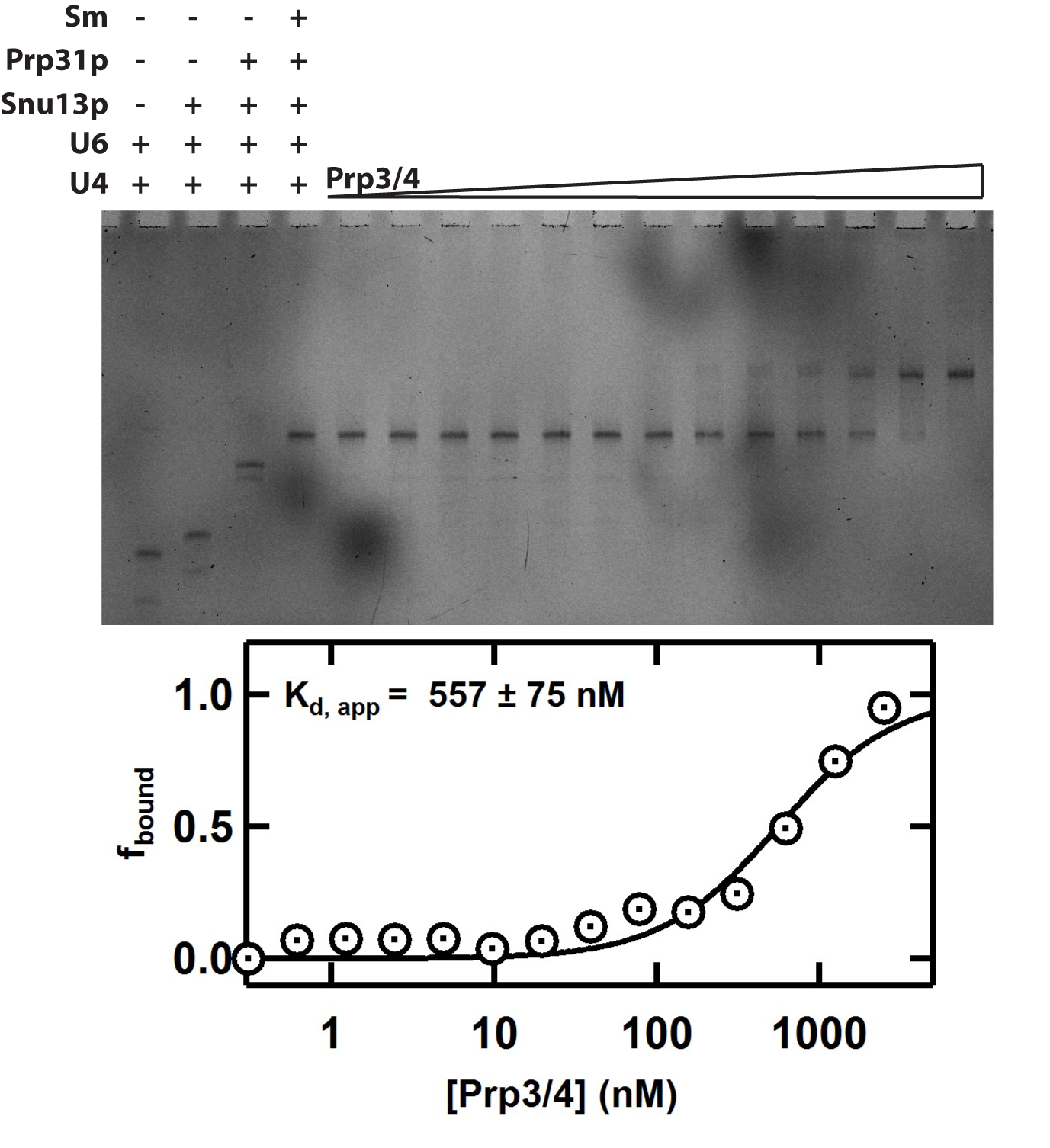
**

**Supplementary Figure 16:** Gel shift and binding curve for the interaction between the pre-formed U4/U6 snRNA/Snu13/Prp31/Sm complex and Prp3/4. The concentrations of pre-formed complex components are U4/U6 snRNA duplex (2 nM), Snu13 (200 nM), Prp31 (120 nM), and Sm proteins (64 nM). The concentration of Prp3/4 ranges from 0-2.5 μM in 2-fold increments. The apparent K_d_ (K_d, app_) is given for a single representative binding curve.


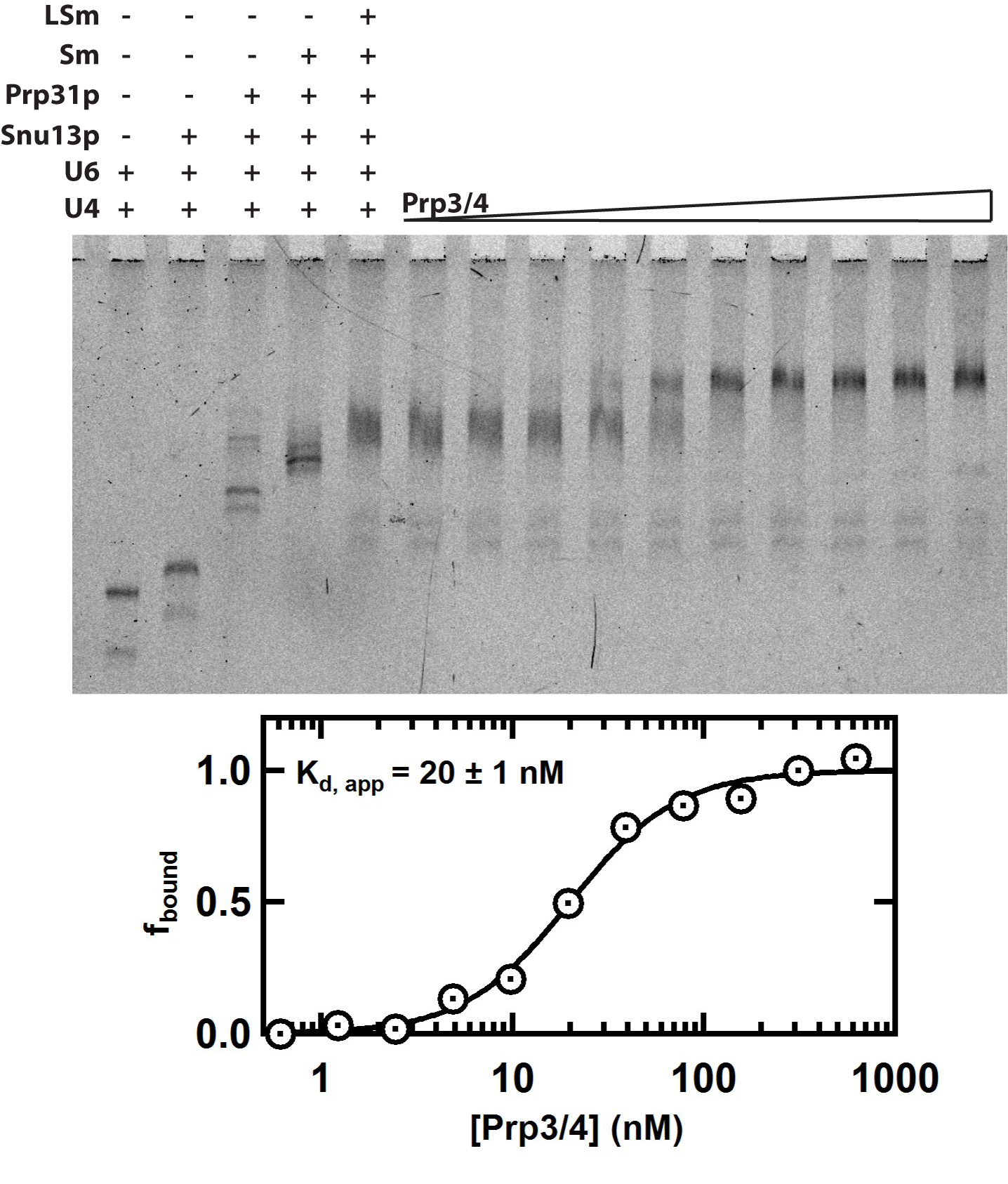


**Supplementary Figure 17:** Gel shift and binding curve for the interaction between the pre-formed U4/U6 snRNA/Snu13/Prp31/Sm/LSm complex and Prp3/4. The concentrations of pre-formed complex components are U4/U6 snRNA duplex (2 nM), Snu13 (200 nM), Prp31 (120 nM), Sm proteins (64 nM), and LSm proteins (240 nM). The concentration of Prp3/4 ranges from 0-2.5 μM in 2-fold increments. The apparent K_d_ (K_d, app_) is given for a single representative binding curve.


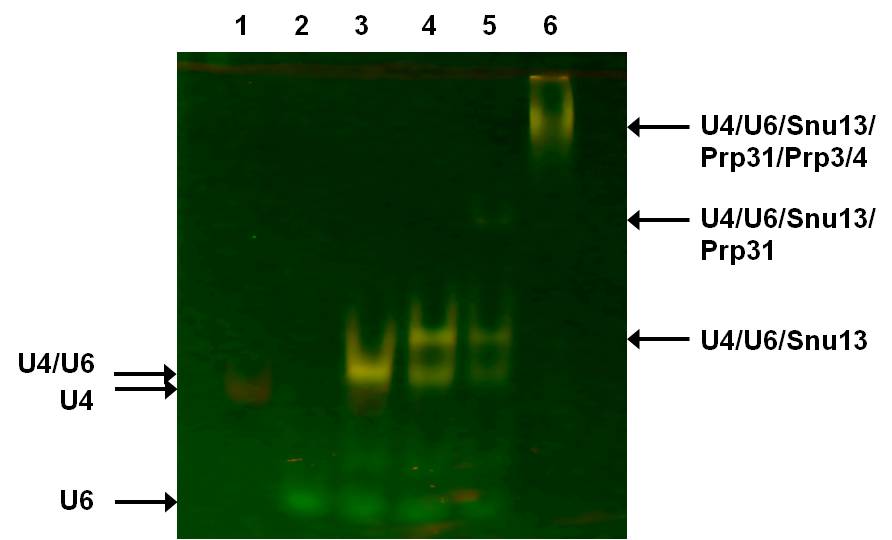


**Supplementary Figure 18:** Minimal fluorophore labeled U4/U6 snRNA duplex binds Snu13, Prp31 and Prp3/4. RNA-protein complex formation followed by EMSA with fluorescence detection. Gel was scanned by exciting with 532 nm and 635 nm lasers to detect Cy3-U6 and Cy5-U4, respectively. Resulting image is an overlay of both channels. Lane 1: Cy5-U4 snRNA (red band). Lane 2: Cy3-U6 snRNA (green band). Lane 3: U4/U6 snRNA duplex (yellow band). Lane 4: U4/U6 snRNA/Snu13 (shifted yellow band). Lane 5: U4/U6 snRNA/Snu13/Prp31 (further shifted band). Lane 6: U4/U6 snRNA/Snu13/Prp31/Prp3/4 (top band). The complete snRNP complex forms in presence of all proteins.


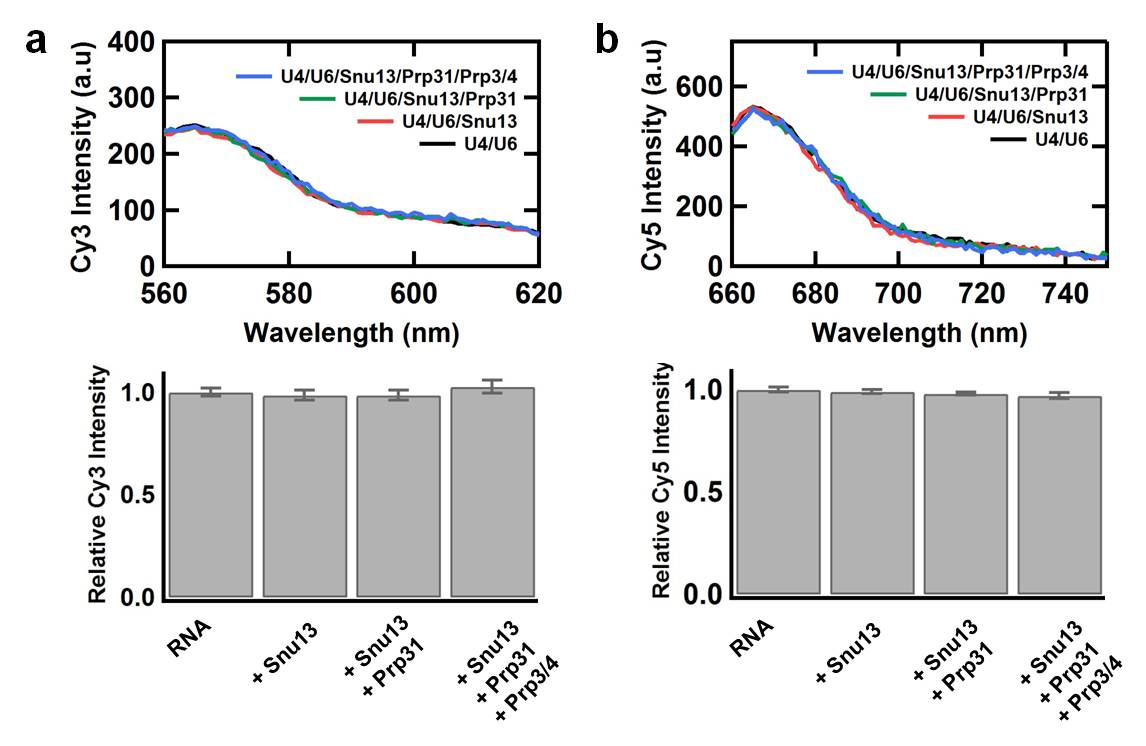


**Supplementary Figure 19:** Fluorescent intensities of Cy3 and Cy5 do not change in the presence of protein. Fluorescence emission spectra of (a) Cy3 and (b) Cy5 the U4/U6 RNA duplex (black) and in presence of Snu13 (red), Snu13 + Prp31 (green), Snu13 + Prp31 + Prp3/4 (blue). The spectra remain unchanged, indicating that the proteins do not affect the fluorescence properties of Cy3 and Cy5.


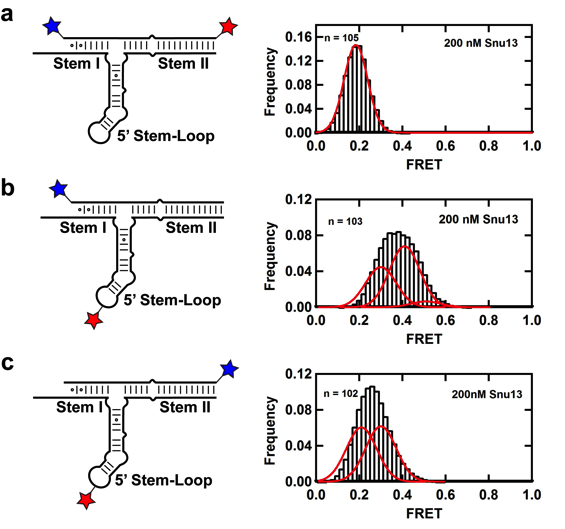


**Supplementary Figure 20:** Single-molecule experiments at elevated concentrations of Snu13 (200 nM) with (a) the stem I-stem II construct, (b) the stem I-5’ Stem-loop construct, and (c) the stem II-5’ stem-loop construct.


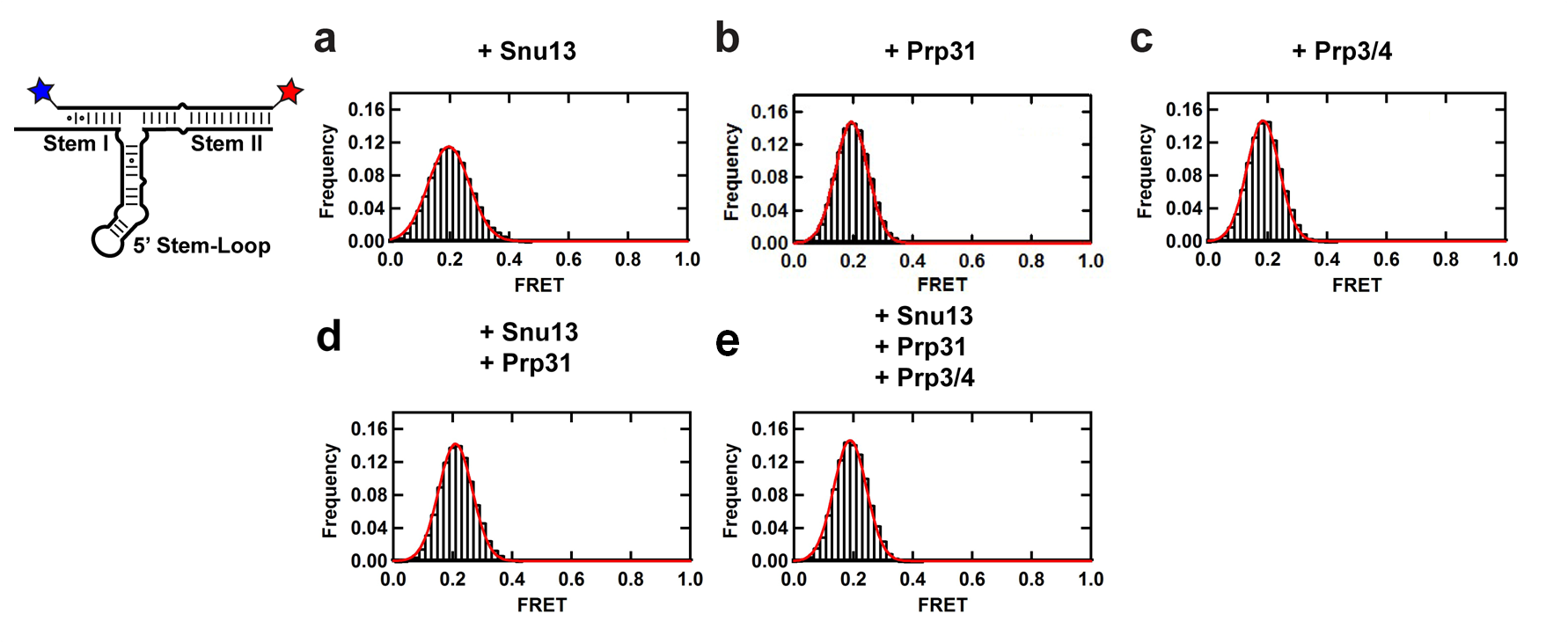


**Supplementary Figure 21:** Stem I and II remain coaxially stacked in presence of any of the snRNP proteins. Single-molecule FRET experiments with the stem I-II construct with (a) Snu13 (b) Prp31 (c) Prp3/4 (d) Snu13+ Prp31 (e) Snu13+ Prp31+ Prp3/4.


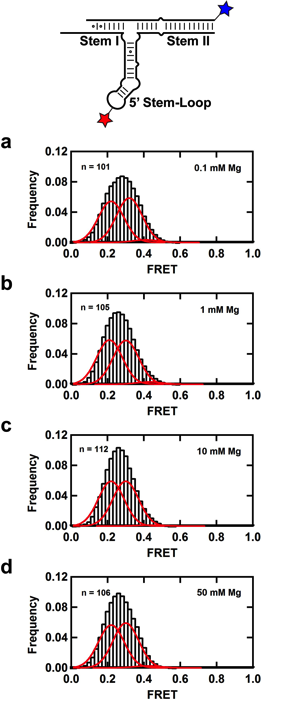


**Supplementary Figure 22:** The U4/U6 snRNA 3-way junction conformation does not change in the presence of Magnesium. Single-molecule FRET experiments with the stem II-5’ stem-loop construct with (a) 0.1 mM Mg (b) 1 mM Mg (c) 10 mM Mg and (d) 50 mM Mg.


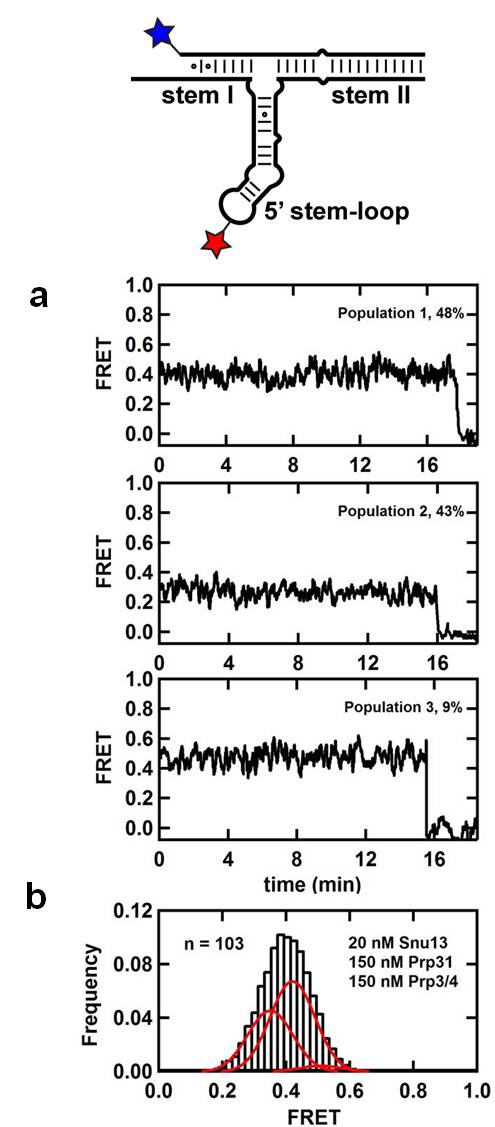


**Supplementary Figure 23:** The snRNP complex remains static for long times. (a) Single-molecule FRET time trajectories and (b) corresponding histogram for experiment recorded for >15 min at 500 ms time resolution with the surface immobilized U4/U6 snRNA duplex in presence of 20 nM Snu13, 150 nM Prp31 and 150 nM Prp3/4. The complex does not exhibit any dynamics within this experimental time window.

**
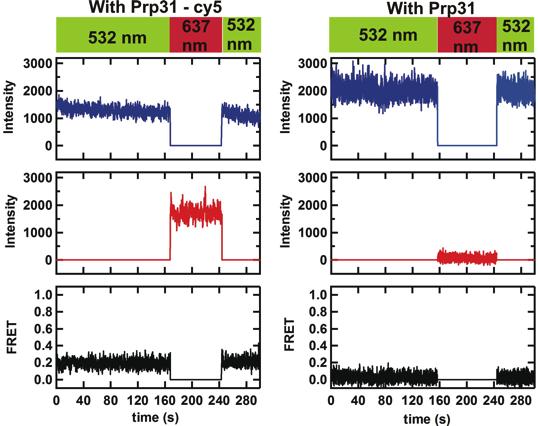
**

**Supplementary Figure 24:** Prp31 protein stays bound to the U4/U6 snRNA duplex even in the presence of Snu13 and Prp3/4. Intensities for the excitation at 532 nm (upper), resulting FRET (lower) and at 637 nm (middle) for the experiment in the presence of Cy5-Prp31 is shown.
